# Differential Use of Human Neutrophil Fc*γ* Receptors for Inducing Neutrophil Extracellular Trap Formation

**DOI:** 10.1155/2016/2908034

**Published:** 2016-01-14

**Authors:** Omar Rafael Alemán, Nancy Mora, Ricarda Cortes-Vieyra, Eileen Uribe-Querol, Carlos Rosales

**Affiliations:** ^1^Departamento de Inmunología, Instituto de Investigaciones Biomédicas, Universidad Nacional Autónoma de México, 04510 México, DF, Mexico; ^2^División de Estudios de Posgrado e Investigación, Facultad de Odontología, Universidad Nacional Autónoma de México, 04510 México, DF, Mexico

## Abstract

Neutrophils (PMN) are the most abundant leukocytes in the blood. PMN migrate from the circulation to sites of infection, where they are responsible for antimicrobial functions. PMN use phagocytosis, degranulation, and formation of neutrophil extracellular traps (NETs) to kill microbes. NETs are fibers composed of chromatin and neutrophil-granule proteins. Several pathogens, including bacteria, fungi, and parasites, and also some pharmacological stimuli such as phorbol 12-myristate 13-acetate (PMA) are efficient inducers of NETs. Antigen-antibody complexes are also capable of inducing NET formation. However the particular Fc*γ* receptor involved in triggering this function is a matter of controversy. In order to provide some insight into what Fc*γ* receptor is responsible for NET formation, each of the two human Fc*γ* receptors was stimulated individually by specific monoclonal antibodies and NET formation was evaluated. Fc*γ*RIIa cross-linking did not promote NET formation. Cross-linking other receptors such as integrins also did not promote NET formation. In contrast Fc*γ*RIIIb cross-linking induced NET formation similarly to PMA stimulation. NET formation was dependent on NADPH-oxidase, PKC, and ERK activation. These data show that cross-linking Fc*γ*RIIIb is responsible for NET formation by the human neutrophil.

## 1. Introduction

Neutrophils (PMN) are the most abundant leukocytes in the blood. PMN are innate immune cells that migrate from the circulation to sites of infection, where they are responsible for antimicrobial functions [1]. PMN use phagocytosis, degranulation, and formation of neutrophil extracellular traps (NETs) to kill microbes [2, 3]. NETs are formed through a unique cell death program named “NETosis” that involves activation in most cases of nicotinamide adenine dinucleotide phosphate- (NADPH-) oxidase, which produces reactive oxygen species (ROS) [4–6]. During NETosis, the characteristic lobular nucleus of neutrophils disappears, and the chromatin expands in the cytosol, while the cell membrane remains intact. Three or four hours after stimulation, the cell membrane breaks and the chromatin fibers get released forming a netlike structure outside the cell. NET fibers are composed of chromatin covered with histones [7] and antimicrobial proteins derived from the neutrophil granules, such as the bactericidal/permeability-increasing protein (BPI), elastase, myeloperoxidase, lactoferrin, and metalloprotease 9 [2, 4]. The requirements for NADPH-oxidase and myeloperoxidase in NET formation differ depending on the stimulus [8, 9]. Besides their antimicrobial capacity, NETs seem to act as a physical barrier where microorganisms get trapped and consequently prevent further spread of pathogens. Thus, NETs bind, block, and kill microorganisms extracellularly and independently of phagocytosis [10].

Binding of receptors for the Fc portion of antibodies (Fc receptors) to opsonized microorganisms is one of the most important mechanisms for pathogen recognition and activation of neutrophils [11]. Human neutrophils express constitutively two antibody receptors that are members of the Fc receptor family for IgG molecules, namely, Fc*γ*RIIa (CD32a) and Fc*γ*RIIIb (CD16b) [12]. These receptors are exclusive human receptors. Fc*γ*RIIa is composed of a single polypeptide chain bearing an ITAM on its cytoplasmic domain [11]. This ITAM confers on Fc*γ*RIIa the ability to initiate signaling events that regulate cell responses, including phagocytosis, cytokine production, antibody-dependent cell-mediated cytotoxicity, and the respiratory burst [13]. Fc*γ*RIIIb is present exclusively on neutrophils and it is a glycophosphatidylinositol- (GPI-) linked receptor, lacking transmembrane and cytoplasmic domains [14]. Although signaling molecules associated with Fc*γ*RIIIb are still unknown and its signaling mechanism remains unidentified, several reports show that Fc*γ*RIIIb can initiate signaling events leading to calcium transients [15], actin polymerization [16], activation of integrins [17], and NF-*κ*B activation [18].

Several pathogens, including virus, bacteria, fungi, and parasites, have all been reported to be inducers of NET formation [10]. In addition, proinflammatory stimuli such as lipopolysaccharide (LPS) [19], interleukin- (IL-) 8, and tumor necrosis factor (TNF) [20, 21] and also some pharmacological stimuli such as phorbol 12-myristate 13-acetate (PMA), an activator of protein kinase C (PKC), are efficient inducers of NETs [2]. Some reports indicate that NET formation was increased when microorganisms were opsonized with autologous serum [3]. This suggested a possible role for Fc*γ*R in NET formation.

Recent reports indicated that antigen-antibody complexes seem capable of inducing NET formation [22, 23]. In one report, a B cell-deficient mouse was used to show NETs could not be formed, thus suggesting a direct role for Fc receptors in this function [23]. In this study, however no particular receptor was identified. In another report, soluble immune complexes were used to activate neutrophils and induce NET formation. It was determined that Fc*γ*RIIIb promoted endocytosis of soluble immune complexes and that Fc*γ*RIIa promoted NET formation in vivo [22]. However, more recently, it was reported that Fc*γ*RIIIb is the receptor responsible for NET formation in response to immobilized immune complexes [24]. Thus, in order to provide some insight into this controversy, each of the two human Fc*γ* receptors was stimulated individually by specific monoclonal antibodies and NET formation was evaluated.

Fc*γ*RIIa cross-linking did not promote NET formation. Cross-linking other receptors such as integrins also did not promote NET formation. In contrast Fc*γ*RIIIb cross-linking induced efficient NET formation similarly to PMA stimulation. NET formation was dependent on NADPH-oxidase, PKC, and extracellular signal-regulated kinase (ERK) activation. These data support the idea that different Fc receptors promote independent cell functions.

## 2. Materials and Methods

### 2.1. Neutrophils

Neutrophils (PMN) were purified from blood collected from adult healthy volunteers exactly as previously described [25]. All volunteers provided a written informed consent for their participation in this study. The informed consent form and all experimental procedures were approved by the Bioethics Committee at Instituto de Investigaciones Biomédicas, UNAM.

### 2.2. Reagents

Bovine serum albumin (BSA) was from F. Hoffmann-La Roche Ltd. (Mannheim, Germany). Piceatannol, a spleen tyrosine kinase (Syk) inhibitor, was from Acros Organics (New Jersey, USA). Wortmannin, a phosphatidylinositol 3-kinase (PI-3K) inhibitor; GÖ6976, a protein kinase C (PKC) inhibitor; GÖ6983, another PKC inhibitor; 3-(1-methyl-1H-indol-3-yl-methylene)-2-oxo-2,3-dihydro-1H-indole-5-sulfonamide (iSyk), another Syk inhibitor (catalog number 574711); and the antibleaching mounting medium FluorSave (catalog number 345789) were from Calbiochem/EMD Millipore (Billerica, MA). UO126, a MEK (ERK kinase) inhibitor, was from Promega (Madison, WI, USA). Diphenyleneiodonium (DPI), an NADPH-oxidase inhibitor; (E)-3-[4-methylphenylsulfonyl]-2-propenenitrile (BAY 117082), an NF-*κ*B inhibitor; phorbol 12-myristate 13-acetate (PMA); and all other chemicals were from Sigma Aldrich (St. Louis, MO). The following antibodies were used: anti-human Fc*γ*RIIa (CD32) mAb IV.3 [26] (ATCC HB-217) was from American Type Culture Collection (Manassas, VA). Anti-*β*1 integrin mAb TS2/16 was donated by Martin Hemler (Dana Farber Cancer Research Institute, Boston, MA). Anti-*β*2 integrin (CD18) mAb IB4 and anti-human Fc*γ*RIIIb (CD16) mAb 3G8 [27] were donated by Dr. Eric J. Brown (University of California in San Francisco, San Francisco, CA). Mouse monoclonal anti-neutrophil elastase (D-7; catalog number sc-365950), rabbit polyclonal anti-histone H2B (FL-126; catalog number sc-10808), rabbit polyclonal anti-ERK 1 (catalog number sc-94), and mouse monoclonal anti-phospho-ERK 1 (pTyr204) (catalog number sc-7383) were from Santa Cruz Biotechnology (Santa Cruz, CA). Alexa Fluor 555-conjugated donkey anti-rabbit IgG (catalog number A-31572), Alexa Fluor 488-conjugated donkey anti-mouse IgG (catalog number A-21202), and FITC-conjugated F(ab′)_2_ goat anti-mouse IgG (catalog number A-10683) were from Invitrogen Molecular Probes (Eugene, OR). F(ab′)_2_ goat anti-mouse IgG (catalog number 0855468), HRP-conjugated F(ab′)_2_ goat anti-mouse IgG (catalog number 0855572), and HRP-conjugated F(ab′)_2_ goat anti-rabbit IgG (catalog number 0855686) were from MP Biomedicals (Santa Ana, CA).

### 2.3. Preparation of Specific Monoclonal Antibodies

Hybridoma cells were grown in DMEM (Gibco; Grand Island, NY) containing 10% fetal bovine serum (FBS) also from Gibco (Grand Island, NY). Antibodies were purified from saturated (8-day-old) tissue culture supernatants with Protein-G Sepharose 4 Fast Flow from GE Healthcare Bio-Sciences AB (Uppsala, Sweden). After elution from the Sepharose with 0.1 M glycine-HCl, pH 2.7, antibodies were dialyzed against PBS, adjusted to 1 mg/mL, and filter-sterilized. Finally, antibodies were stored in small aliquots at −80°C. The functionality of antibodies was confirmed by their binding to neutrophil receptors (Supplemental Figure 1S in Supplementary Material available online at http://dx.doi.org/10.1155/2016/2908034).

### 2.4. Labeling of Neutrophil Receptors with Specific Monoclonal Antibodies

For receptor stimulation, PMN were treated with specific anti-receptor monoclonal antibodies as follows. PMN (1 × 10^6^) in 500 *μ*L PBS were placed into a 1.5 mL Eppendorf tube, and 10 *μ*g/mL of the corresponding anti-receptor monoclonal antibody was added. Cells were incubated at 4°C for 20 minutes and then washed twice with 1 mL of cold PBS (4°C) centrifuged at 1,743 ×g, 1 minute each time. This centrifugation protocol did not preactivate cells as long as they were maintained cold. Next, PMN were resuspended in 500 *μ*L cold (4°C) RPMI-1640 medium (Gibco; Grand Island, NY) containing 5% fetal bovine serum (FBS) also from Gibco (Grand Island, NY).

### 2.5. NET Formation Assay

Neutrophils were left untreated for PMA stimulation or previously treated with anti-receptor antibodies (as described above) for receptor stimulation. Neutrophils (1 × 10^6^) in 500 *μ*L RPMI-1640 medium were added to each well of a 24-well plate, containing a 12 mm coverslip, and then incubated in a humidified incubator with 5% CO_2_ at 37°C for 30 minutes. Then 100 *μ*L of 120 nM PMA in PBS or 100 *μ*L of 450 *μ*g/mL F(ab′)_2_ goat anti-mouse IgG (for receptor stimulation) was added to each well. Plates were incubated in 5% CO_2_ at 37°C for 4 hours. Next, 600 *μ*L of 2% paraformaldehyde in PBS was added to each well, and the plates were incubated overnight in 5% CO_2_ at 37°C.

In selected experiments, PMN were incubated for 30 minutes before stimulation, with the inhibitors piceatannol (50 *μ*M), wortmannin (50 nM), UO126 (50 *μ*M), GÖ6983 (1 *μ*M), GÖ6976 (1 *μ*M), DPI (10 *μ*M), BAY 117082 (2.5 *μ*M), or the vehicle dimethyl sulfoxide (DMSO) alone.

### 2.6. NET Visualization and Immunofluorescence

All washes and incubations were done at room temperature by placing the coverslip upside down over a 250 *μ*L drop of each solution formed on a well of Parafilm placed on a tube rack, exactly as previously described [28]. Coverslips were taken out of the 24-well plate one at a time and washed four times with water for 5 minutes each. Next, they were placed over 0.1% Triton X-100 in 4% paraformaldehyde for 10 minutes, then on PBS for 5 minutes, and then on 10 *μ*g/mL of the corresponding primary antibody (anti-neutrophil elastase or anti-histone) in 5% BSA in PBS for 60 minutes. Coverslips were then washed four times with PBS for 5 minutes each and placed on 8 *μ*g/mL of the corresponding secondary antibody (Alexa Fluor-conjugated anti-rabbit IgG or anti-mouse IgG) in 5% BSA in PBS containing 300 nM DAPI. Coverslips were incubated in the dark for 60 minutes. Finally, coverslips were mounted on a microscope slide with one drop of FluorSave. Slides were observed with a fluorescence inverted microscope model IX-70 from Olympus (Center Valley, PA). Images were captured with an Evolution-VF Cooled Color camera from Media Cybernetics (Rockville, MD) and the computer program QCapture Pro 6.0 from QImaging, Surrey (British Columbia, Canada). Images were processed with the computer program ImageJ 1.47v from the National Institutes of Health (Bethesda, MD).

### 2.7. NET Formation with Opsonized Particles

Opsonization of 4.8 *μ*m fluorescent (catalog number 16592) or nonfluorescent (catalog number 17135) latex particles from Polysciences, Inc. (Warrington, PA), was performed exactly as described [29]. These particles were used in phagocytosis assays as described [30] or in NET formation assays as follows. PMN (1 × 10^6^ cells) in 500 *μ*L were centrifuged in a 1.5 mL Eppendorf tube at 1,743 ×g for 1 min. After removing the supernatant, the cell pellet was disaggregated by tapping the tube against a rack, and 80 *μ*L of opsonized latex particles (1.25 × 10^8^ particles/mL) resuspended in RPMI-1640 medium with 5% FBS was added. The PMN-particle mixture was incubated at 4°C for 20 min. Then, 1 mL of cold PBS was added, the tube was centrifuged at 1,743 ×g for 1 min, and the cell pellet was resuspended in 500 *μ*L RPMI-1640 medium with 5% FBS. Cell suspension was transferred into a well of a 24-well plate, containing a 12 mm coverslip, and then incubated in a humidified incubator with 5% CO_2_ at 37°C for 4 h. Then 500 *μ*L of 2% paraformaldehyde in PBS was added to each well, and the plate was kept in the incubator overnight. Finally, the coverslip was used for NET visualization as described above.

### 2.8. Quantification of NETs

A 96-well plate was previously covered with 25 *μ*g/mL poly-D-lysine for three hours at room temperature. Each well was then washed three times with 50 *μ*L PBS for 5 minutes each time, and the plate was allowed to air dry inside a flow laminar hood for two hours. Neutrophils were resuspended at 1.25 × 10^6^ cells/mL in RPMI-1640 medium, containing 500 nM SYTOX Green (Molecular Probes, Inc.; Eugene, OR), and 80 *μ*L of this cell suspension (1 × 10^5^ PMN) was added to each well of the 96-well plate. Next, the plate was incubated at 37°C in a 5% CO_2_ incubator for 20 minutes. For Fc*γ*R stimulation, the supernatant was removed gently with a micropippetor and 50 *μ*L of 10 *μ*g/mL of the corresponding anti-Fc receptor antibody was added to each well. The plate was placed in a 35°C prewarmed microplate reader model Synergy HT from BioTek Instruments (Winooski, VT) and incubated there for 20 minutes. Next, the supernatant was removed gently with a micropippetor and 100 *μ*L of 75 *μ*g/mL of F(ab′)_2_ goat anti-mouse IgG containing 500 nM SYTOX Green was added to each corresponding well. At this time, for PMA stimulation 20 *μ*L of 100 nM PMA was added to each corresponding well. The plate was then incubated for up to 4 hours. For this assay, cells were not fixed. Finally the fluorescence from the bottom of the plate was read, using the 485 nm excitation and 528 emission filters.

For NET formation induced with opsonized latex particles, PMN (1 × 10^6^ cells) in 500 *μ*L RPMI-1640 medium with 5% FBS and 500 nM SYTOX Green were mixed with 80 *μ*L of opsonized latex particles (1.25 × 10^8^ particles/mL). Then 100 *μ*L of the PMN-particle mixture was transferred into a well of a 96-well plate and incubated in a 35°C prewarmed microplate reader for 4 hours. Fluorescence from the bottom of the plate was read using the 485 nm excitation and 528 emission filters.

### 2.9. Western Blotting

Western blots were performed exactly as previously described [31]. Cells were lysed in RIPA buffer (150 mM NaCl, 5 mM EDTA, 50 mM HEPES, 0.5% sodium deoxycholate, 1% Nonidet P-40, and 10 mM 2-mercaptoethanol, pH 7.5) containing cOmplete protease inhibitor cocktail from Roche (Basel, Switzerland), for 15 minutes at 4°C. Cell lysates were then cleared by centrifugation and proteins resolved on SDS 12% PAGE. Proteins were then electrotransferred onto polyvinylidene fluoride (PVDF) membranes (Immobilon-P; Millipore, Bedford, MA). Membranes were incubated in blocking buffer (1% BSA and 5% nonfat dry milk (Carnation; Nestle, Glendale, CA) and 0.1% Tween 20 in PBS) overnight at 4°C. Membranes were subsequently probed with the corresponding antibody in blocking buffer, for 1 hour at room temperature, anti-ERK 1 (1/1000 dilution) or anti-phospho-ERK 1 (1/500 dilution). Membranes were washed with PBS six times and incubated with a 1/3000 dilution of HRP-conjugated F(ab′)_2_ goat anti-rabbit IgG or HRP-conjugated F(ab′)_2_ goat anti-mouse IgG for 1 hour at room temperature. After washing six more times, the membrane was developed with a chemiluminescence substrate (SuperSignal; Pierce, Rockford, IL) according to the manufacturer's instructions.

### 2.10. Determination of Apoptosis

Apoptosis was assayed with the FITC annexin V and propidium iodide (PI) dead cell apoptosis kit for flow cytometry (catalog number V13242) from Molecular Probes, Inc. (Eugene, OR), following the manufacturer's instructions. Briefly, PMN were treated with nothing, PMA, or the antibodies against each of the Fc*γ* receptors as described above. After a two-hour incubation at 37°C, PMN (1 × 10^5^) were washed in PBS and resuspended in annexin-binding buffer (10 mM HEPES, 140 mM NaCl, and 2.5 mM Ca^2+^, pH 7.4), and 2 *μ*L of FITC annexin V and 1 *μ*L of 1.5 mM PI were added. Cells were incubated for 15 min at room temperature and then 400 *μ*L of annexin-binding buffer was added. Cells were immediately analyzed by flow cytometry. PMN apoptosis (positive control) was induced by UV-light irradiation as previously described [32].

### 2.11. Reactive Oxygen Species (ROS)

ROS production was assessed with the DCFDA-Cellular Reactive Oxygen Species Detection Assay Kit (catalog number ab113851) from Abcam, Inc. (Cambridge, MA), following the manufacturer's instructions. Briefly, PMN were treated with mAb IV.3 or mAb 3G8 at 10 *μ*g/mL for 20 minutes on ice. PMN were washed with 1x buffer and then incubated with 15 *μ*M DCFDA in 1x buffer for 30 minutes at 37°C. After one wash in 1x buffer, 5 × 10^4^ PMN were placed in each well of a 96-well clear-bottom black plate from Corning Inc. (New York, NY) and incubated for 20 minutes at 35°C in a plate reader, model Synergy HT from BioTek Instruments (Winooski, VT). Then, for antibody treatment 50 *μ*L of goat anti-mouse IgG (150 *μ*g/mL) was added and for PMA treatment, 50 *μ*L of PMA (40 nM) was added. Fluorescence was read for two hours at excitation 485 nm and emission 535 nm.

### 2.12. Phagocytosis Assays

Neutrophil phagocytosis was determined as previously described [29]. Briefly, PMN (1 × 10^5^ cells) were resuspended in 100 *μ*L cold phagocytosis buffer (2 mM calcium chloride, 1.5 mM magnesium chloride, and 1% human serum albumin in PBS) and mixed with 3.5 *μ*L of a suspension (1 × 10^8^ beads/mL) of IV.3-opsonized, or 3G8-opsonized or control-opsonized (no antibody) fluorescent latex beads. PMN and beads were incubated at 37°C for 30 min, centrifuged at 6000 rpm for 1 min, and resuspended in 100 *μ*L of ice-cold trypsin-EDTA solution (0.05% trypsin, 1 mM EDTA in PBS) to detach uninternalized beads from the cells. After a 15 min incubation on ice, PMN were washed with 1 mL cold PBS plus 0.5% BSA plus 2 mM EDTA and resuspended in 500 *μ*L cold 1% paraformaldehyde in PBS. To analyze phagocytosis by flow cytometry, latex particles were gated out during sample acquisition and 10,000 cells were acquired per sample. Phagocytosis was reported as percent of fluorescence-positive cells (cells internalizing at least one fluorescent particle). Phagocytosis was also analyzed by microscopy and reported as phagocytic index, the number of beads internalized by 100 cells.

### 2.13. Statistical Analysis

Quantitative data were expressed as mean ± standard error of mean (SEM). Single variable data were compared by unpaired-sample Student's *t*-tests using the computer program KaleidaGraph version 3.6.2 for Mac (Synergy Software; Reading, PA). Also, variance homogeneity was checked by using Levene's test, and multiple pair-comparisons were performed using Tukey's test after ordinary one-way analysis of variance (ANOVA) [33]. Post hoc differences were considered statistically different at a value *p* < 0.05. Analyses were done using the SAS software version 9.0 (2012) from SAS Institute Inc. (Cary, NC).

## 3. Results

Several types of pathogens have been reported to induce NET formation, but there are not reports on particular receptors used by neutrophils to recognize these pathogens and to induce NETosis. Most studies on NETs have used PMA, a potent activator of PKC, and efficient inducer of NETs [2]. In this case, no receptor is involved since PMA directly activates intracellular signaling. Some reports indicated that NET formation was increased when microorganisms were opsonized with autologous serum and also that antigen-antibody complexes seemed to be capable of inducing NET formation. These studies suggested a possible role for IgG Fc receptors (Fc*γ*R) in NET formation. However the particular Fc*γ* receptor involved in triggering this function is a matter of controversy. Thus, in order to explore what particular Fc receptor could induce NET formation, PMN were stimulated by cross-linking individual receptors with specific monoclonal antibodies.

**Figure 1:**
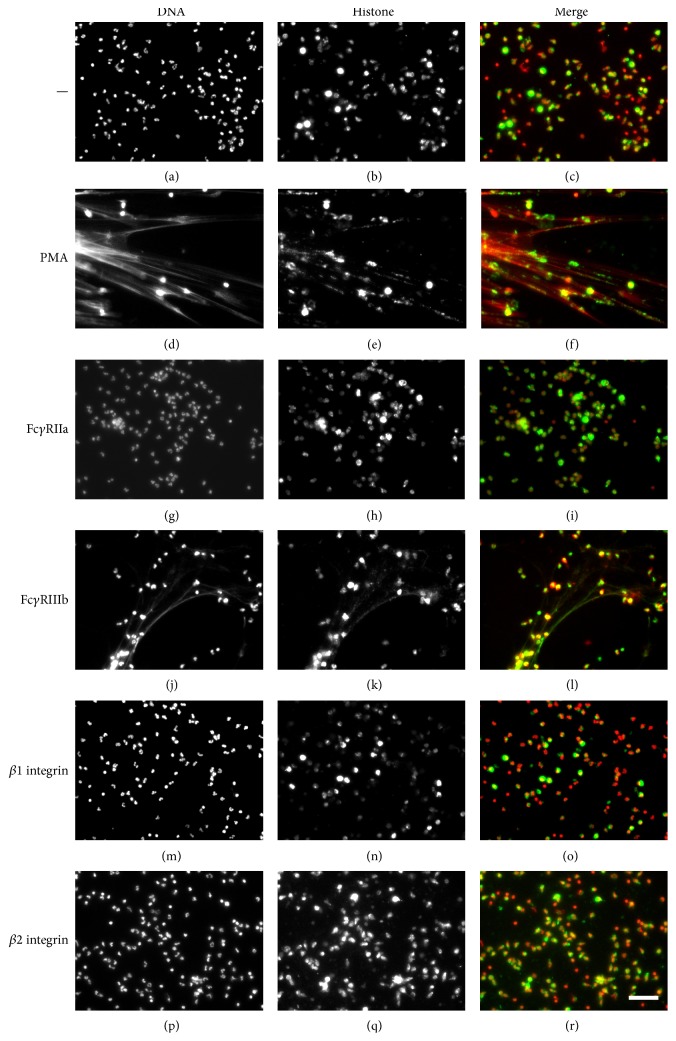
Fc*γ*RIIIb induces NET formation. (a) Human neutrophils (PMN) were left untreated (—) or were stimulated with 20 nM phorbol 12-myristate 13-acetate (PMA), by cross-linking Fc*γ*RIIa with mAb IV.3, by cross-linking Fc*γ*RIIIb with mAb 3G8, by cross-linking *β*1 integrins with mAb TS2/16, or by cross-linking *β*1 integrins with mAb IB4. After four hours, PMN were fixed and stained for DNA (DAPI, red) and for histone (green). Microphotographs were taken at 200x magnification and are representative of more than 10 experiments. Bar is 50 *μ*m.

**Figure 2:**
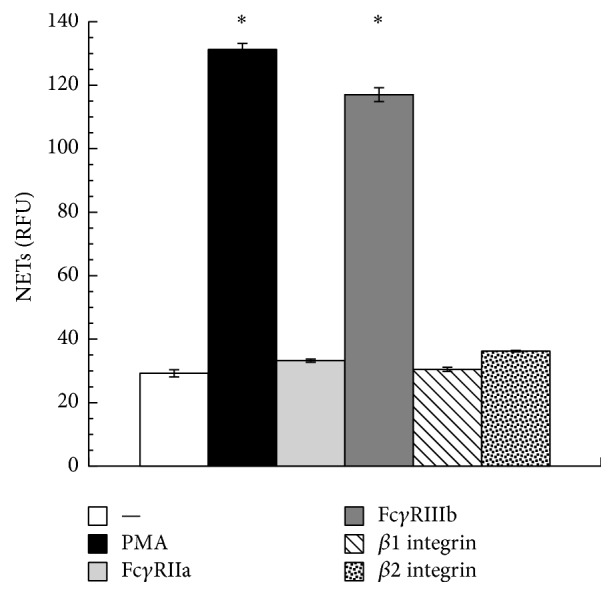
NETs are induced by PMA and Fc*γ*RIIIb cross-linking. Human neutrophils (PMN) were left untreated (—) or were stimulated with 20 nM phorbol 12-myristate 13-acetate (PMA), by cross-linking Fc*γ*RIIa with mAb IV.3, by cross-linking Fc*γ*RIIIb with mAb 3G8, by cross-linking *β*1 integrins with mAb TS2/16, or by cross-linking *β*2 integrins with mAb IB4. The relative amount of NETs, as extracellular DNA of nonfixed cells, was estimated from SYTOX Green fluorescence in relative fluorescent units (RFU) at 4 hours after stimulation, as described in Materials and Methods. Data are mean ± SEM of 4 experiments. Asterisks denote conditions that are statistically different from control (*p* < 0.05).

**Figure 3:**
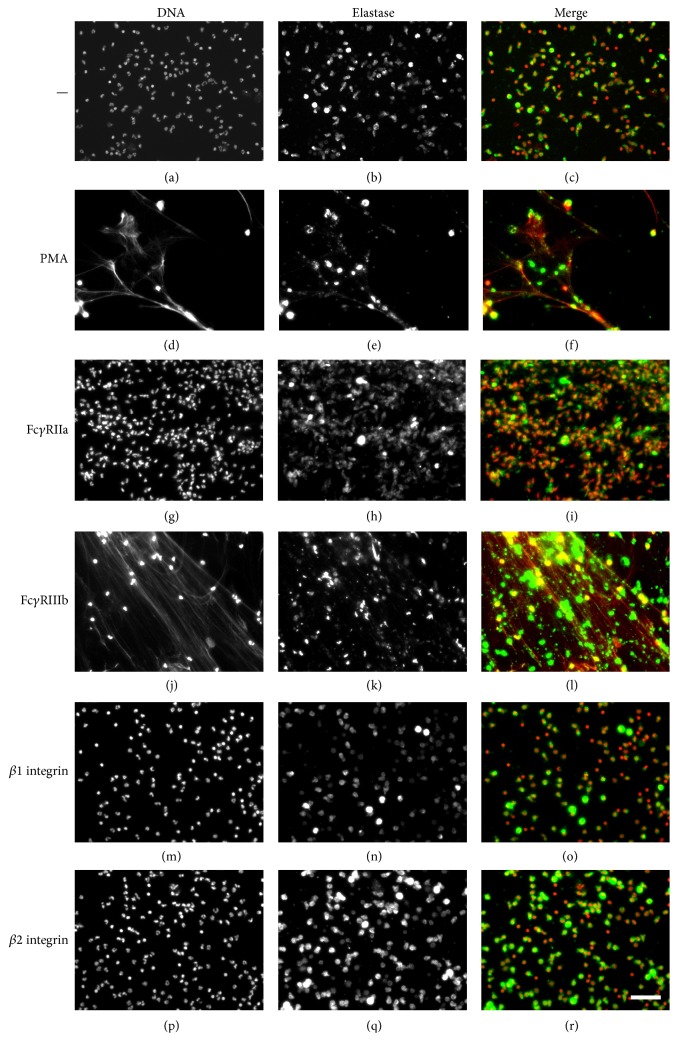
NETs are decorated with neutrophil elastase. Human neutrophils (PMN) were stimulated with 20 nM phorbol 12-myristate 13-acetate (PMA), by cross-linking Fc*γ*RIIa with mAb IV.3, by cross-linking Fc*γ*RIIIb with mAb 3G8, by cross-linking *β*1 integrins with mAb TS2/16, or by cross-linking *β*1 integrins with mAb IB4. After four hours, PMN were fixed and stained for DNA (DAPI, red) or for elastase (green). Microphotographs were taken at 200x magnification and are representative of five experiments. Bar is 50 *μ*m.

When PMN were stained with DAPI, the typical lobular nuclei were clearly seen (Figure 1(a)). Immunolabeling of histones also showed the localization of these proteins within the PMN nucleus (Figure 1(b)). When PMN were treated with PMA, nuclei lost their typical morphology and long NETs were formed (Figure 1(d)). Also, the cell morphology was altered; PMN appeared larger and diffuse (Supplemental Figure 2S). Histones were also present along the extracellular DNA fibers (Figure 1(e)). Cross-linking Fc*γ*RIIa with the specific mAb IV.3 did not induce NET formation and PMN retained intact nuclei with typical lobular morphology (Figures 1(g) and 1(h)). Similarly, cross-linking *β*1 integrins (Figures 1(m) and 1(n)) or *β*2 integrins (Figures 1(p) and 1(q)) did not induce any NET formation (Supplemental Figure 2S). In contrast, cross-linking Fc*γ*RIIIb with the specific mAb 3G8 induced strong NET formation (Figure 1(j)) similar to the one induced by PMA (Figure 2). These Fc*γ*RIIIb-induced extracellular DNA fibers were also covered with histones (Figure 1(k) and Supplemental Figure 3S). Cross-linking of Fc*γ*RIIa together with Fc*γ*RIIIb or *β*2 integrins together with Fc*γ*RIIIb did not induce changes in the amount of NET formation induced by Fc*γ*RIIIb alone (not shown). An important characteristic of NETs is that they are covered with antimicrobial proteins from the PMN granules. The presence of neutrophil elastase on NETs was confirmed for NETs induced both by PMA and by Fc*γ*RIIIb (Figure 3 and Supplemental Figure 4S). These data indicated that cross-linking Fc*γ*RIIIb is an efficient stimulus for NET formation. NETosis [4] is a form of cell death different from apoptosis [34]. In neutrophils, apoptosis appears spontaneously when these cells get older or after they have activated their proinflammatory functions [35]. Because the NET quantification method is related to detection of extracellular DNA, it was important to determine whether PMN were in apoptosis after stimulation of Fc*γ* receptors. After PMA stimulation or Fc*γ* receptor cross-linking, PMN did not have an increase in annexin V-binding, indicating that PMN were not in apoptosis [34, 36] (Supplemental Figure 5S).

**Figure 4:**
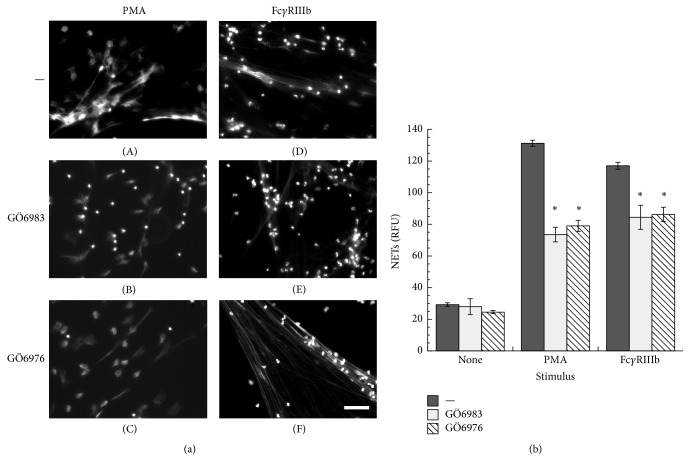
Fc*γ*RIIIb-induced NET formation is dependent on PKC. (a) Human neutrophils (PMN) were stimulated with 20 nM phorbol 12-myristate 13-acetate (PMA) or by cross-linking Fc*γ*RIIIb with mAb 3G8. PMN were previously treated with solvent alone (—) or with the PKC inhibitors GÖ6983 (1 *μ*M) or GÖ6976 (1 *μ*M). After four hours, PMN were fixed and stained for DNA (DAPI). Microphotographs were taken at 200x magnification and are representative of five experiments. Bar is 50 *μ*m. (b) The relative amount of NETs was estimated from SYTOX Green fluorescence in relative fluorescent units (RFU) at 4 hours after stimulation. Data are mean ± SEM of 11 experiments. Asterisks denote conditions that are statistically different from control (*p* < 0.001).

**Figure 5:**
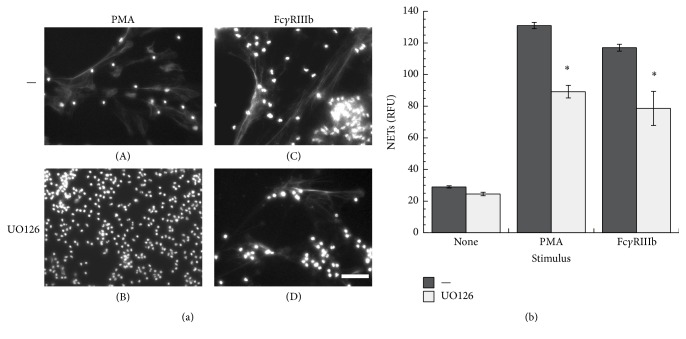
Fc*γ*RIIIb-induced NET formation is dependent on MEK. (a) Human neutrophils (PMN) were not stimulated (None) or were stimulated with 20 nM phorbol 12-myristate 13-acetate (PMA) or by cross-linking Fc*γ*RIIIb with mAb 3G8. PMN were previously treated with solvent alone (—) or with the MEK inhibitor UO126 (50 *μ*M). After four hours, PMN were fixed and stained for DNA (DAPI). Microphotographs were taken at 200x magnification and are representative of five experiments. Bar is 50 *μ*m. (b) The relative amount of NETs was estimated from SYTOX Green fluorescence in relative fluorescent units (RFU) at 4 hours after stimulation. Data are mean ± SEM of 8 experiments. Asterisks denote conditions that are statistically different from control (*p* < 0.001).

**Figure 6:**
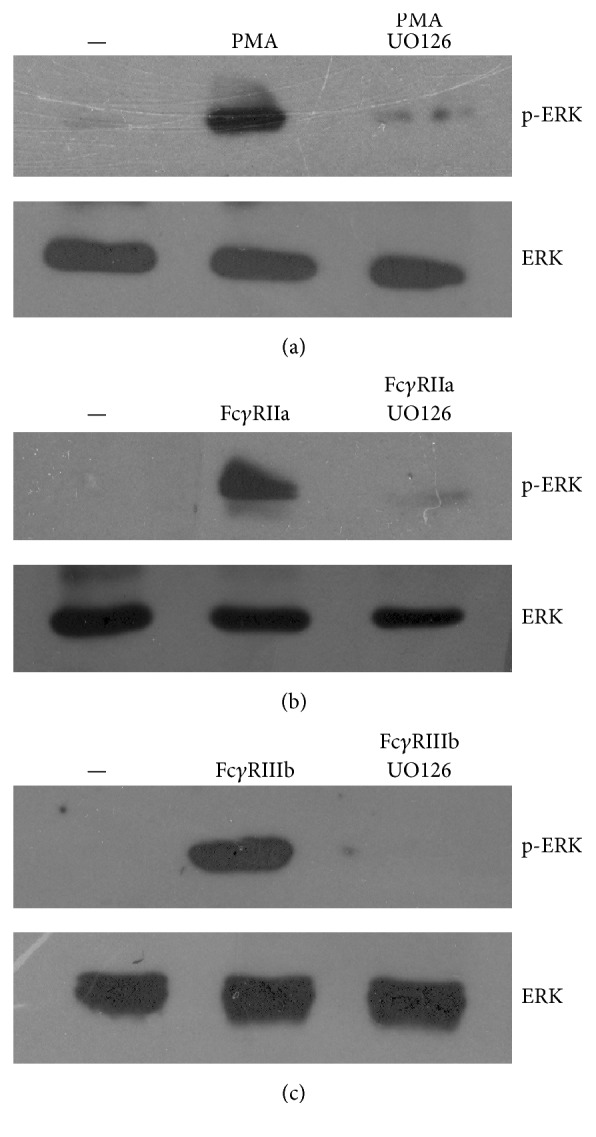
Fc*γ*R-cross-linking induces activation of ERK. Human neutrophils (PMN) were left untreated (—) or were stimulated (a) with 20 nM phorbol 12-myristate 13-acetate (PMA) or (b) by cross-linking Fc*γ*RIIa with mAb IV.3 or (c) by cross-linking Fc*γ*RIIIb with mAb 3G8. PMN were also stimulated in the presence of the MEK inhibitor UO126 (50 *μ*M). PMN cell lysates were prepared after 30 min stimulation. Proteins were resolved by SDS-PAGE and then Western blotted for phosphorylated-ERK (p-ERK) (upper panel) or for total ERK (lower panel). Data are representative of three separate experiments.

Because PMA is an activator of PKC, the involvement of this kinase in NET formation induced by Fc*γ*RIIIb was tested with two specific PKC inhibitors. PMN treated with PMA formed NETs as expected (Figure 4). However, when PMN were treated previously with GÖ6983, an inhibitor of PKC*α*, PKC*β*, and PKC*γ* isozymes (Figure 4), or with GÖ6976, a conventional PKC inhibitor (Figure 4), NETs were not formed after PMA stimulation. Similarly, NET formation after Fc*γ*RIIIb cross-linking (Figure 4) was inhibited by these PKC inhibitors (Figure 4). In addition, downstream of PKC, the MEK, ERK pathway has been reported to participate in NET formation after PMA stimulation [37]. When PMN were treated with UO126, a potent specific MEK inhibitor, NETs were not formed after PMA stimulation (Figure 5). Also, UO126 treatment blocked NET formation after Fc*γ*RIIIb stimulation (Figure 5). These data suggested that Fc*γ*RIIIb stimulation led to NET formation using PKC and ERK. To confirm that ERK 1 was activated after PMA or Fc receptor stimulation as previously reported [38], PMN were stimulated in the presence or absence of the MEK inhibitor and ERK 1 activation was detected by Western blotting. PMA induced ERK phosphorylation in PMN (Figure 6(a)), and this ERK activation was completely blocked by the MEK inhibitor UO126 (Figure 6(a)). Similarly, Fc*γ*RIIa cross-linking (Figure 6(b)) or Fc*γ*RIIIb cross-linking (Figure 6(c)) resulted in efficient ERK 1 phosphorylation. This ERK 1 activation was completely blocked in both cases by the MEK inhibitor (Figures 6(b) and 6(c)). These data suggested that both Fc*γ* receptors can induce ERK activation, but this enzyme is not sufficient for NET formation, since only Fc*γ*RIIIb led to release of NETs.

**Figure 7:**
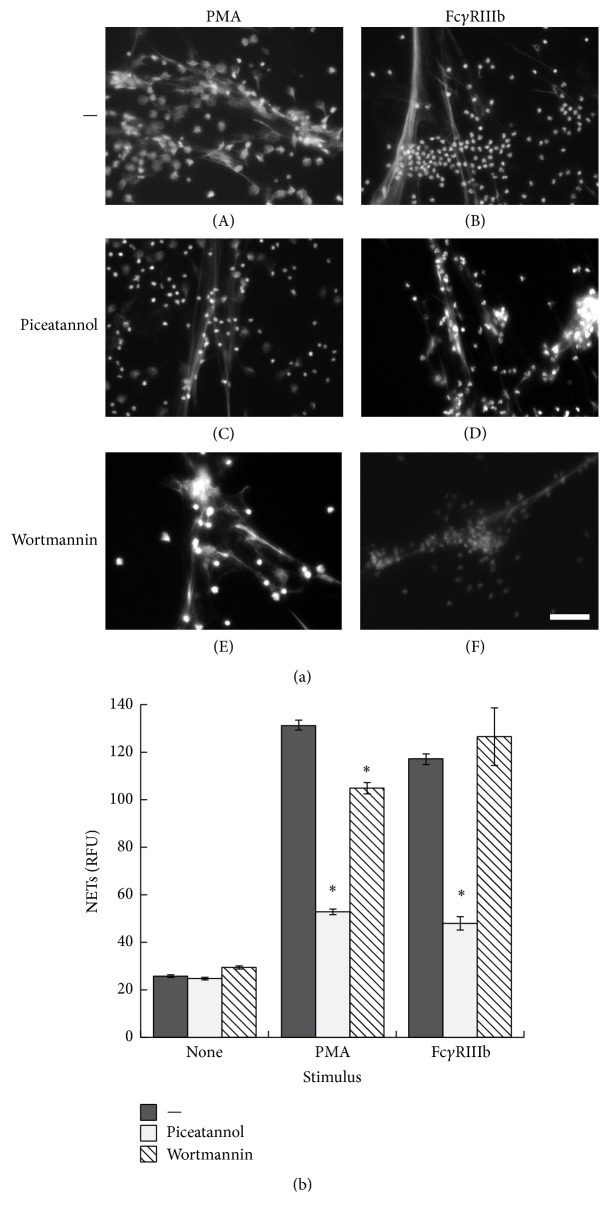
Fc*γ*RIIIb-induced NET formation requires Syk but is independent of PI-3K. (a) Human neutrophils (PMN) were not stimulated (None) or were restimulated with 20 nM phorbol 12-myristate 13-acetate (PMA) or by cross-linking Fc*γ*RIIIb with mAb 3G8. PMN were previously treated with solvent alone (—) or with the Syk inhibitor piceatannol (50 *μ*M) or with the PI-3K inhibitor wortmannin (50 nM). After four hours, PMN were fixed and stained for DNA (DAPI). Microphotographs were taken at 200x magnification and are representative of three experiments. Bar is 50 *μ*m. (b) The relative amount of NETs was estimated from SYTOX Green fluorescence in relative fluorescent units (RFU) at 4 hours after stimulation. Data are mean ± SEM of 5 experiments. Asterisks denote conditions that are statistically different from control (*p* < 0.05).

Other signaling molecules that are important for Fc receptor signaling via ITAM are Syk and PI-3K. Although, Fc*γ*RIIIb does not have an ITAM, it has been suggested that Fc*γ*RIIIb might signal in cooperation with Fc*γ*RIIa [39]. Thus, to explore the possible involvement of these molecules in Fc*γ*RIIIb-induced NET formation, PMN were treated with piceatannol (Figure 7) or iSyk (Supplemental Figure 6S), selective inhibitors of Syk, or with wortmannin (Figure 7), a selective inhibitor of PI-3K, before stimulation. These inhibitors prevented NET formation when PMN were stimulated by PMA (Figure 7). Similarly, NET formation was inhibited after cross-linking of Fc*γ*RIIIb in the presence of piceatannol (Figure 7) but proceeded normally in the presence of wortmannin (Figure 7). Interestingly, iSyk only caused small but statistically significant inhibition of NET formation after PMA stimulation, while it did not block Fc*γ*RIIIb-induced NET formation (Supplemental Figure 6S). These data suggested that Fc*γ*RIIIb-induced NET formation involves Syk, but it is independent of PI-3K.

**Figure 8:**
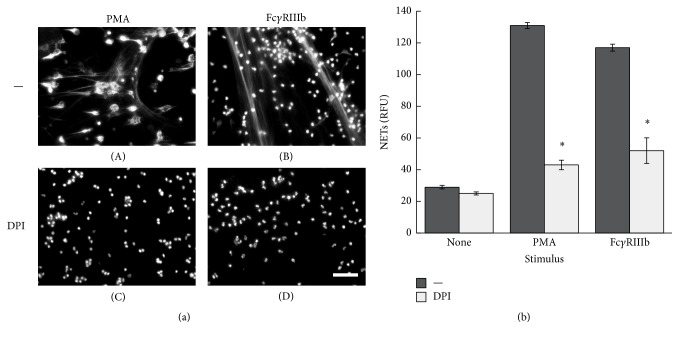
Fc*γ*RIIIb-induced NET formation is dependent on NADPH-oxidase. (a) Human neutrophils (PMN) were not stimulated (None) or were stimulated with 20 nM phorbol 12-myristate 13-acetate (PMA) or by cross-linking Fc*γ*RIIIb with mAb 3G8. PMN were previously treated with solvent alone (—) or with the NADPH-oxidase inhibitor diphenyleneiodonium (DPI) (10 *μ*M). After four hours, PMN were fixed and stained for DNA (DAPI). Microphotographs were taken at 200x magnification and are representative of three experiments. Bar is 50 *μ*m. (b) The relative amount of NETs was estimated from SYTOX Green fluorescence in relative fluorescent units (RFU) at 4 hours after stimulation. Data are mean ± SEM of 5 experiments. Asterisks denote conditions that are statistically different from control (*p* < 0.001).

**Figure 9:**
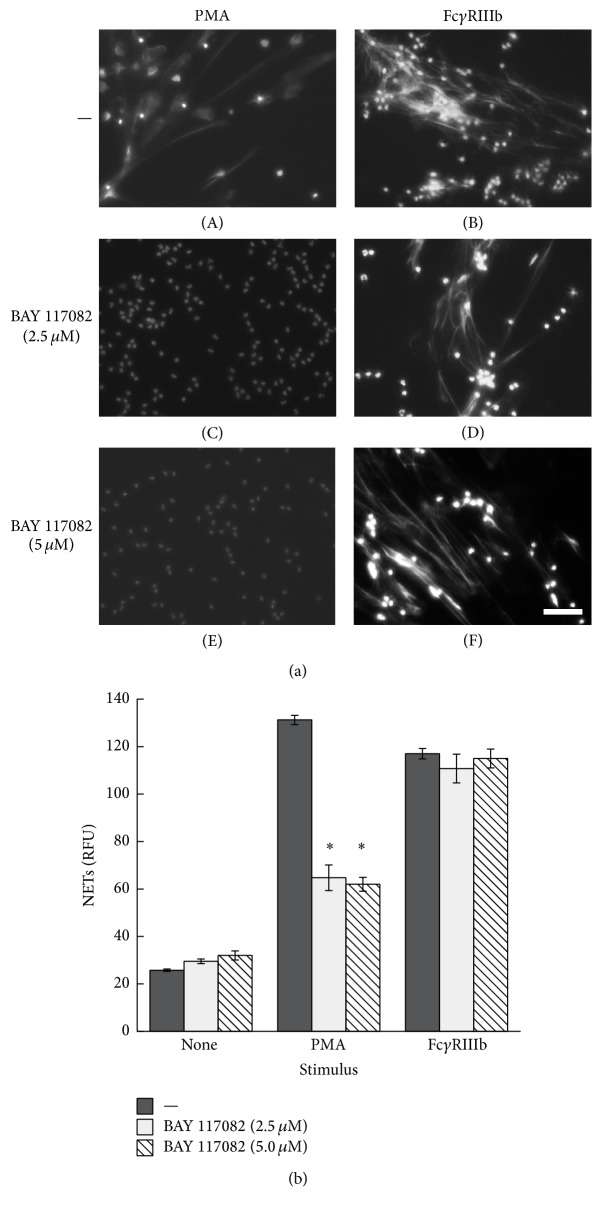
Fc*γ*RIIIb-induced NET formation is independent of NF-*κ*B. (a) Human neutrophils (PMN) were not stimulated (None) or were stimulated with 20 nM phorbol 12-myristate 13-acetate (PMA) or by cross-linking Fc*γ*RIIIb with mAb 3G8. PMN were previously treated with solvent alone (—) or with the NF-*κ*B inhibitor BAY 117082 at 2.5 *μ*M and 5 *μ*M. After four hours, PMN were fixed and stained for DNA (DAPI). Microphotographs were taken at 200x magnification and are representative of three experiments. Bar is 50 *μ*m. (b) The relative amount of NETs was estimated from SYTOX Green fluorescence in relative fluorescent units (RFU) at 4 hours after stimulation. Data are mean ± SEM of 4 experiments. Asterisks denote conditions that are statistically different from control (*p* < 0.05).

NETs formed after PMA stimulation require activation of NADPH-oxidase and formation of ROS [40] and also activation of NF-*κ*B [41]. Thus, we explored the involvement of these molecules in Fc*γ*RIIIb-induced NET formation. PMN treated with diphenyleneiodonium (DPI), an NADPH-oxidase inhibitor, were not able to form NETs after PMA stimulation (Figure 8). Similarly, DPI-treated PMN could not form NETs after cross-linking of Fc*γ*RIIIb (Figure 8). PMA treatment, as well as cross-linking of both Fc*γ*RIIa and Fc*γ*RIIIb, indeed induced ROS production that was completely blocked by DPI (Supplemental Figure 7S). In addition, PMN treated with BAY 117082, an NF-*κ*B inhibitor, were not able to form NETs after PMA stimulation (Figure 9). In contrast, PMN treated with BAY 117082 at two different concentrations formed NETs efficiently after cross-linking of Fc*γ*RIIIb (Figure 9). These data suggested that Fc*γ*RIIIb could indeed induce the formation of NETs via NADPH-oxidase activation, but independently of NF-*κ*B activation.

**Figure 10:**
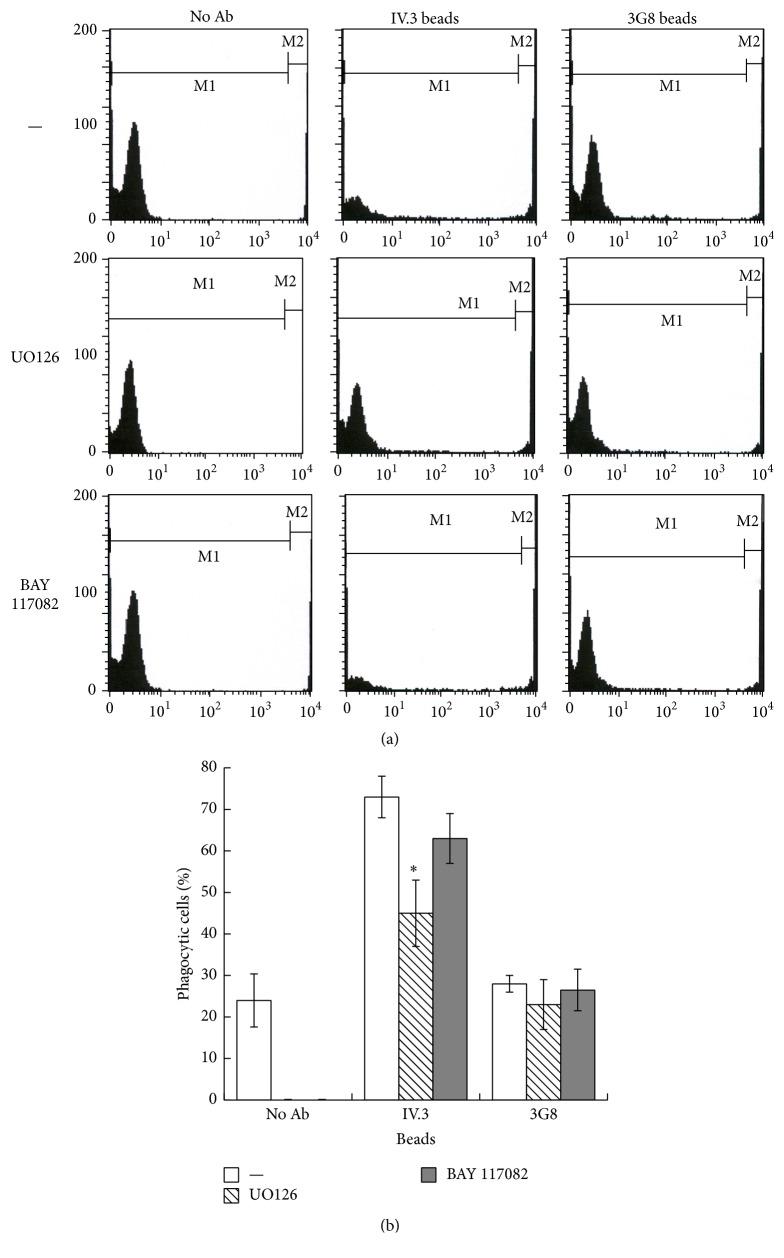
Fc*γ*RIIa induces efficient phagocytosis. (a) Human neutrophils were mixed with fluorescence latex particles. Particles were nonopsonized (no Ab) or opsonized with monoclonal antibody (mAb) IV.3 anti-Fc*γ*RIIa or with mAb 3G8 anti-Fc*γ*RIIIb. Cells were allowed to ingest the particles for 30 min. Phagocytosis of latex beads was also evaluated in the absence (None) or the presence of 50 *μ*M UO126 (MEK inhibitor) or 2.5 *μ*M BAY 117082 (NF-*κ*B inhibitor). Phagocytosis was assessed by flow cytometry, detecting the reduced number of cells with low fluorescence (M1 marker), and the appearance of cells with high fluorescence in the far right side (M2 marker) of the histogram of gated PMN. (b) Phagocytosis was quantified by flow cytometry, as the percentage of high-fluorescence cells (marker M2) in the histogram of gated PMN. Data are representative of four separate experiments.

**Figure 11:**
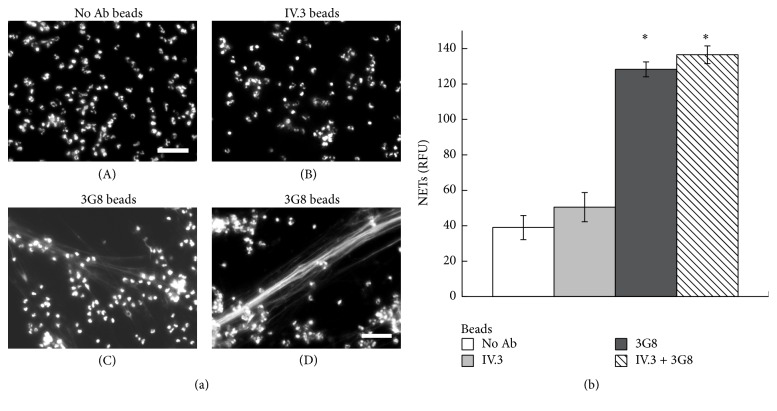
Anti-Fc*γ*RIIIb-opsonized particles induce NET formation. (a) Human neutrophils (PMN) mixed with latex particles nonopsonized (no Ab beads) or opsonized with monoclonal antibody IV.3, anti-Fc*γ*RIIa (IV.3 beads), or with monoclonal antibody 3G8, anti-Fc*γ*RIIIb (3G8 beads), were incubated for four hours and fixed and stained for DNA (DAPI). Microphotographs were taken at 200x magnification and are representative of three experiments. Bar is 50 *μ*m. (b) The relative amount of NETs was estimated from SYTOX Green fluorescence in relative fluorescent units (RFU) at 4 hours after stimulation, as described in Materials and Methods. Data are mean ± SEM of four experiments. Asterisks denote conditions that are statistically different from control (*p* < 0.02).

Clearly, selective activation of Fc*γ*RIIIb on the PMN membrane was enough to induce the formation of NETs. In order to explore whether cross-linking of Fc receptors by a more natural stimulus could also induce NET formation, PMN were mixed with opsonized latex particles. These particles covered with Protein A and then opsonized with selective anti-Fc receptor antibodies can be recognized by only one or the other of the Fc receptors. As shown previously [30], PMN were capable of efficient phagocytosis of latex beads opsonized with mAb IV.3 (anti-Fc*γ*RIIa) and of very poor phagocytosis of latex beads opsonized with mAb 3G8 (anti-Fc*γ*RIIIb) (Figure 10). These beads were opsonized at similar levels with both anti-Fc*γ* receptor antibodies (Supplemental Figure 8S). PMN and fluorescent beads can be easily separated as two distinct populations in a flow cytometer. Thus, by gating on cells an increase in fluorescence indicates efficient phagocytosis (Supplemental Figure 9S). The efficient Fc*γ*RIIa-mediated phagocytosis was dependent on ERK activation [30], since the MEK inhibitor UO126 prevented it (Figure 10), and it was independent of NF-*κ*B activation, since the inhibitor BAY 117082 did not affect it (Figure 10). These data were also confirmed by evaluating phagocytosis by microscopy (Supplemental Figure 10S). In contrast, the poor phagocytic response of Fc*γ*RIIIb was independent of both MEK and NF-*κ*B (Figure 11). These beads when not opsonized (Figure 11(a)(A)) or when opsonized with anti-Fc*γ*RIIa antibodies (Figure 11(a)(B)) could not induce the formation of NETs. However, beads opsonized with anti-Fc*γ*RIIIb antibodies efficiently induced the formation of NETs (Figure 11(a)(C and D)). In addition, a mixture of beads opsonized with either anti-Fc*γ*RIIa antibodies or anti-Fc*γ*RIIIb antibodies also induced NET formation to the same level as anti-Fc*γ*RIIIb beads alone (Figure 11(b)). These data strongly suggested that Fc*γ*RIIa can efficiently promote phagocytosis, while it cannot induce the formation of NETs. In contrast, Fc*γ*RIIIb poorly promotes phagocytosis, but it can efficiently induce the formation of NETs.

## 4. Discussion

Neutrophils are the most abundant circulating leukocytes in mammals and they are rapidly recruited to sites of infection, where they act as the first line of defense against invading pathogens [42]. Neutrophil activation, through various membrane receptors, is also required for the initiation of the several defense mechanisms displayed by these cells [43], including phagocytosis, respiratory burst, release of various microbicidal molecules by degranulation [44], and the recently described formation of neutrophil extracellular traps (NETs) [3]. NETs are extracellular fibers formed by chromatin covered with histones [7] and antimicrobial proteins derived from neutrophil granules [2]. NETs seem to act as a physical barrier where microorganisms get trapped [10] and display antimicrobial activity that is independent of phagocytosis [45]. Despite the fact that many pathogens, including virus, bacteria, fungi, and parasites [10], have all been reported to induce NET formation, no particular receptors on the neutrophil membrane leading to release of NETs have been identified until very recently. IgA-opsonized bacteria or IgA-opsonized beads activated the Fc*α*RI (CD89) leading to release of NETs [46]. Other previous reports indicated that NET formation was increased when microorganisms were opsonized with autologous serum [3], and also antigen-antibody complexes seemed capable of inducing NET formation [22, 23]. These reports thus suggested a role for IgG Fc receptors (Fc*γ*Rs) in NET formation.

In the neutrophil two types of Fc*γ*R are constitutively expressed, namely, Fc*γ*RIIa and Fc*γ*RIIIb [12, 13]. This fact has made it difficult to establish which functions are initiated by each of these two Fc*γ*Rs. For phagocytosis, there is no doubt that Fc*γ*RIIa is an important receptor [30]. In contrast, Fc*γ*RIIIb is an important receptor for signaling to the nucleus [38]. In the case of NET formation, it is not clear which Fc*γ*R is preferentially responsible for this function. It was previously reported that Fc*γ*RIIIb promoted endocytosis of soluble immune complexes and that Fc*γ*RIIa promoted NET formation in vivo [22]. However, more recently, it was reported that Fc*γ*RIIIb is the receptor responsible for NET formation in response to immobilized immune complexes [24]. In addition, neutrophil stimulation by IgG antineutrophil cytoplasmic antibodies (ANCA) led to degranulation and neutrophil extracellular trap formation in an Fc*γ*RIIIb allele-specific manner [47]. Here, we have found that indeed Fc*γ*RIIIb, but not Fc*γ*RIIa, induced significant amounts of NETs.

Selective Fc*γ*RIIIb cross-linking with specific monoclonal antibodies on human neutrophils induced NET formation. The release of these NETs was detected 3-4 hours after stimulation and was dependent on ROS, since the NADPH-oxidase inhibitor DPI abrogated trap release. This NET release was similar to the one induced by cross-linking Fc*α*RI [46] or by phorbol 12-myristate 13-acetate (PMA) stimulation [2] but different from the rapid, oxidant-independent NET release recently described [48]. Fc*γ*RIIIb-induced NETs consisted of long DNA fibers decorated with histones and neutrophil elastase showing a bona fide neutrophil extracellular trap structure. Although both Fc*γ*RIIa and Fc*γ*RIIIb induced a strong respiratory burst as shown by activated ROS production, cross-linking of Fc*γ*RIIa alone did not induce NET formation. ROS are required for NET formation in most cases [2, 4, 8], but they are not sufficient, since ROS production induced by phagocytosis cannot initiate NET formation [9].

Fc*γ*RIIIb-induced NETs are similar in shape and molecular structure to those induced by PMA [9], and the molecular mechanism leading to their release seems to be also similar. Most studies on NET formation have been conducted with PMA stimulation [2]. PMA is a direct activator of protein kinase C (PKC); thus any possible receptor involved in NET formation is bypassed. Several inhibitors of PKC have been shown to block NET formation [49]. In agreement with those reports, we found that two different inhibitors of PKC indeed blocked NET formation after PMA and Fc*γ*RIIIb stimulation. In addition, inhibition of Syk with piceatannol blocked the release of NETs induced either by PMA or by Fc*γ*RIIIb (Figure 7). However, inhibition of Syk with iSyk slightly reduced only PMA-induced NETosis (Figure 6S). The differential inhibition of NET formation with two reported Syk inhibitors suggests that some of the discrepancies in the literature regarding signaling pathways regulating NETosis may be due to the use of various pharmacological inhibitors. It is necessary to revise these pathways more carefully in future studies. Despite this caveat, inhibition of Syk with piceatannol points to an important role for this kinase in NET formation induced by specific cross-linking of Fc*γ*RIIIb. Syk was also found to participate in NET formation induced by soluble immune complexes [22, 50], by insoluble immune complexes [24], and by PMA [24]. Syk is normally associated with initial signaling events at the level of cell surface receptors, but PMA can bypass these receptors to directly activate PKC [51]. Yet activation of Syk by PMA has been previously described in neutrophils. PMA induced PKC-dependent phosphorylation of Syk [52], and piceatannol reduced ROS production in response to PMA [53]. Together these reports and our data support the idea that Syk activation is involved in both PMA- and also Fc*γ*RIIIb-induced ROS-dependent NETosis.

Downstream of PKC, a role for the MEK, ERK pathway [54] and for NF-*κ*B [41] in PMA-induced NET formation has been suggested. MEK inhibition blocked PMA- and Fc*γ*RIIIb-induced NETosis indicating that ERK activation is required in this process. ERK was also found to be required for NET formation in response to soluble immune complexes [22] and immobilized immune complexes [24]. However, the role of ERK in NET formation remains unclear. A previous report indicated that ERK is required for NADPH-oxidase activation [37], placing ERK upstream of ROS production, while another report suggested that ROS are downstream of ERK activation [54]. Therefore, it seems that NADPH-oxidase activation for NET formation may proceed not only through an ERK pathway, but also independently of ERK activation, depending on the stimulus [19, 20]. As previously reported, NF-*κ*B inhibition reduced PMA-induced NET formation [41]. However, Fc*γ*RIIIb-induced NETosis was unaffected when neutrophils were treated with the same inhibitor for NF-*κ*B (Figure 9). Similarly, inhibition of PI-3K by wortmannin reduced NET formation by PMA but had no effect on Fc*γ*RIIIb-induced NET formation (Figure 7). A possible role for PI-3K involvement in NET formation induced by immobilized immune complexes was also found using the inhibitor LY29004 [24]. We did not find the same result, but as mentioned above for Syk the particular inhibitor used may be responsible for these different results. It was proposed that PI-3K could influence NF-*κ*B activation via phosphatidylinositol-trisphosphate and in turn NF-*κ*B activate genes important for signaling to NET formation [41]. These ideas, however, have not been proven experimentally and the role of PI-3K and NF-*κ*B in Fc*γ*RIIIb-mediated NETosis needs further exploration.

Fc*γ*RIIIb has been suggested to signal in cooperation with other molecules such as integrin Mac-1 (CD11b/CD18), also known as complement receptor 3 [55]. However, complement receptor ligands are not sufficient to induce NET formation in isolated neutrophils [9]. Similarly, in our case selective cross-linking of *β*2 integrins with mAb IB4 also did not induce any NET formation. In contrast, blocking Mac-1 with antibodies against both CD11b and CD18 chains prevented NET formation by LPS [19], by *β*-glucan [56], and by immobilized immune complexes [24]. These reports and our data suggest that *β*2 integrins cooperate with other receptors to induce NETosis, but they cannot by themselves cause NET formation. The involvement of *β*2 integrins in NET formation might be more related to the adhesion requirement of neutrophils to form NETs [28] than to a signaling capacity of the integrin. Along the same line of thought, cross-linking of other receptors such as *β*1 integrins also did not promote any NET formation (Figure 1), although the same procedure was capable of activating NF-*κ*B in neutrophils [25]. Recently, it was also reported that NET formation in response to* Candida albicans* required fibronectin via *β*1 integrins. However, *β*1 integrin engagement alone was not sufficient to activate NETosis [56]. Similarly, the adhesive protein invasin from* Yersinia pseudotuberculosis* promotes bacteria crossing the intestine epithelium by binding to *β*1 integrins on M-cells. Invasin was also shown to induce ROS and NET formation [57]. However, invasin-mediated triggering of *β*1 integrin was essential but not sufficient for NET production [57]. Additional, so far uncharacterized costimuli were required for NET formation. Clearly, integrins cooperate in different scenarios to activate NETosis after various stimuli including immune complexes, but the exact role they play in this process remains elusive.

Moreover, as mentioned above, selective cross-linking of Fc*γ*RIIa also did not promote NET formation. This was not due to a defect in Fc*γ*RIIa signaling because the same cross-linking procedure led to robust activation of ERK (Figure 6). In addition, latex beads opsonized with the specific anti-Fc*γ*RIIa mAb IV.3 were efficiently phagocytosed by neutrophils [30] (Figure 10). The same opsonized latex beads were not capable of inducing any NET formation. In contrast, latex beads opsonized with the specific anti-Fc*γ*RIIIb mAb 3G8 were poorly phagocytosed by neutrophils [30] (Figures 10 and 10S) but efficiently induced NET formation. Our results support the idea presented in a recent report showing that neutrophils sensed microbe size and selectively released NETs in response to large pathogens, such as* Candida albicans* hyphae and extracellular aggregates of* Mycobacterium bovis*, but not in response to small yeast or single bacteria [58]. In this study, phagocytosis via the receptor dectin-1 acted as a sensor of microbe size and prevented NET release by downregulating the translocation of neutrophil elastase to the nucleus [58]. Similarly, we present here that neutrophils responded via Fc*γ*RIIa with efficient phagocytosis; however NET formation was absent. In contrast, stimulation via Fc*γ*RIIIb led to poor phagocytosis but to significant NET formation. Thus we conclude that NETs are not formed when an opsonized target can be efficiently phagocytosed via Fc*γ*RIIa. However, upon inefficient phagocytosis via Fc*γ*RIIIb engagement, NET formation is induced strongly. Together, these data support the idea that indeed each Fc*γ*R on the human neutrophil is capable of triggering specific responses. Fc*γ*RIIa promotes efficient phagocytosis, while Fc*γ*RIIIb induces NET formation instead. The inflammatory environment may be responsible for what receptor Fc*γ*RIIa or Fc*γ*RIIIb may predominate and initiate a particular cell response [11]. Fc*γ*RIIIb is expressed 4- to 5-fold more abundantly and has a higher affinity for IgG than Fc*γ*RIIa [59], thus probably becoming the preferred receptor to first engage immune complexes. At the same time, inflammatory stimuli can lead to Fc*γ*RIIIb shedding from the cell, favoring now immune complex interactions with Fc*γ*RIIa [60] to induce phagocytosis and cytotoxicity [13]. We believe that when a strong activating threshold is achieved by cross-linking Fc*γ*RIIIb an efficient induction of NET formation takes place.

In conclusion, our data show that Fc*γ*RIIIb governs Fc receptor-induced NET formation in human neutrophils. The signaling pathway used by Fc*γ*RIIIb to induce NETs involves PKC, PKC, and ERK 1. Our results also support the idea that different Fc receptors promote independent cell functions.

## Supplementary Material

The Fcgamma Receptor and Integrin expression on human neutrophils was confirmed with use of the corresponding monoclonal antibodies (Fig. 1S). Neutrophils and NETs are shown both in bright field and fluorescence images (Fig. 2S). NETs are decorated with histones (Fig. 3S) and with neutrophil elastase (Fig. 4S) as shown in these higher magnification (400 X) microphotographs. Neutrophils were not in apoptosis after FcgammaR crosslinking since they did not bind Annexin V (Fig. 5S). NETs induced by Fc gamma RIIIb crosslinking was not inhibited by the Syk inhibitor iSyk (Fig. 6S). Reactive Oxygen Species (ROS) formation was inhibited by the NADPH-oxidase inhibitor diphenyleneiodinium (DPI) (Fig. 7S). Fluorescent beads used in phagocytosis experiments were efficiently opsonized by anti FcgammaR-specific antibodies (Fig. 8S), and could be easily separated from neutrophils by flow cytometry (Fig. 9S). Phagocytosis was assessed the the appearance of cells with high fluorescence (Fig. 9S). Phagocytosis was also assessed by microscopy, calculating the Phagocytic Index, the number of beads ingested by 100 neutrophils (Fig. 10S).

## References

[1] Mócsai A. (2013). Diverse novel functions of neutrophils in immunity, inflammation, and beyond. *The Journal of Experimental Medicine*.

[2] Brinkmann V., Reichard U., Goosmann C. (2004). Neutrophil extracellular traps kill bacteria. *Science*.

[3] Yipp B. G., Petri B., Salina D. (2012). Infection-induced NETosis is a dynamic process involving neutrophil multitasking in vivo. *Nature Medicine*.

[4] Fuchs T. A., Abed U., Goosmann C. (2007). Novel cell death program leads to neutrophil extracellular traps. *Journal of Cell Biology*.

[5] Bianchi M., Hakkim A., Brinkmann V. (2009). Restoration of NET formation by gene therapy in CGD controls aspergillosis. *Blood*.

[6] Remijsen Q., Berghe T. V., Wirawan E. (2011). Neutrophil extracellular trap cell death requires both autophagy and superoxide generation. *Cell Research*.

[7] Neeli I., Radic M. (2012). Knotting the NETs: analyzing histone modifications in neutrophil extracellular traps. *Arthritis Research and Therapy*.

[8] Parker H., Dragunow M., Hampton M. B., Kettle A. J., Winterbourn C. C. (2012). Requirements for NADPH oxidase and myeloperoxidase in neutrophil extracellular trap formation differ depending on the stimulus. *Journal of Leukocyte Biology*.

[9] Branzk N., Papayannopoulos V. (2013). Molecular mechanisms regulating NETosis in infection and disease. *Seminars in Immunopathology*.

[10] Papayannopoulos V., Zychlinsky A. (2009). NETs: a new strategy for using old weapons. *Trends in Immunology*.

[11] Rosales C., Uribe-Querol E. (2013). Antibody—Fc receptor interactions in antimicrobial functions. *Current Immunology Reviews*.

[12] Unkeless J. C., Shen Z., Lin C.-W., DeBeus E. (1995). Function of human Fc*γ*RIIA and Fc*γ*RIIIB. *Seminars in Immunology*.

[13] Rosales C., Uribe-Querol E. (2013). Fc receptors: cell activators of antibody functions. *Advances in Bioscience and Biotechnology*.

[14] Selvaraj P., Rosse W. F., Silber R., Springer T. A. (1988). The major Fc receptor in blood has a phosphatidylinositol anchor and is deficient in paroxysmal nocturnal haemoglobinuria. *Nature*.

[15] Marois L., Paré G., Vaillancourt M., Rollet-Labelle E., Naccache P. H. (2011). Fc*γ*RIIIb triggers raft-dependent calcium influx in IgG-mediated responses in human neutrophils. *The Journal of Biological Chemistry*.

[16] Salmon J. E., Browle N. L., Edberg J. C., Kimberly R. P. (1991). Fc*γ* receptor III induces actin polymerization in human neutrophils and primes phagocytosis mediated by Fc*γ* receptor II. *The Journal of Immunology*.

[17] Ortiz-Stern A., Rosales C. (2005). Fc*γ*RIIIB stimulation promotes *β*1 integrin activation in human neutrophils. *Journal of Leukocyte Biology*.

[18] García-García E., Rosales C. (2007). Nuclear factor activation by Fc*γ*R in human peripheral blood neutrophils detected by a novel flow cytometry-based method. *Journal of Immunological Methods*.

[19] Neeli I., Dwivedi N., Khan S., Radic M. (2009). Regulation of extracellular chromatin release from neutrophils. *Journal of Innate Immunity*.

[20] Neeli I., Khan S. N., Radic M. (2008). Histone deimination as a response to inflammatory stimuli in neutrophils. *The Journal of Immunology*.

[21] Gupta A. K., Joshi M. B., Philippova M. (2010). Activated endothelial cells induce neutrophil extracellular traps and are susceptible to NETosis-mediated cell death. *FEBS Letters*.

[22] Chen K., Nishi H., Travers R. (2012). Endocytosis of soluble immune complexes leads to their clearance by Fc*γ*RIIIB but induces neutrophil extracellular traps via Fc*γ*RIIA in vivo. *Blood*.

[23] Short K. R., von Köckritz-Blickwede M., Langereis J. D. (2014). Antibodies mediate formation of neutrophil extracellular traps in the middle ear and facilitate secondary pneumococcal otitis media. *Infection and Immunity*.

[24] Behnen M., Leschczyk C., Möller S. (2014). Immobilized immune complexes induce neutrophil extracellular trap release by human neutrophil granulocytes via Fc*γ*RIIIB and Mac-1. *Journal of Immunology*.

[25] García-García E., Uribe-Querol E., Rosales C. (2013). A simple and efficient method to detect nuclear factor activation in human neutrophils by flow cytometry. *Journal of Visualized Experiments*.

[26] Looney R. J., Abraham G. N., Anderson C. L. (1986). Human monocytes and U937 cells bear two distinct Fc receptors for IgG. *The Journal of Immunology*.

[27] Fleit H. B., Wright S. D., Unkeless J. C. (1982). Human neutrophil Fc*γ* receptor distribution and structure. *Proceedings of the National Academy of Sciences of the United States of America*.

[28] Brinkmann V., Laube B., Abed U. A., Goosmann C., Zychlinsky A. (2010). Neutrophil extracellular traps: how to generate and visualize them. *Journal of Visualized Experiments*.

[29] García-García E., Brown E. J., Rosales C. (2007). Transmembrane mutations to Fc*γ*RIIA alter its association with lipid rafts: implications for receptor signaling. *The Journal of Immunology*.

[30] Rivas-Fuentes S., García-García E., Nieto-Castañeda G., Rosales C. (2010). Fc*γ* receptors exhibit different phagocytosis potential in human neutrophils. *Cellular Immunology*.

[31] Reyes-Reyes M., Mora N., Zentella A., Rosales C. (2001). Phosphatidylinositol 3-kinase mediates integrin-dependent NF-*κ*B and MAPK activation through separate signaling pathways. *Journal of Cell Science*.

[32] Sweeney J. F., Nguyen P. K., Omann G. M., Hinshaw D. B. (1997). Ultraviolet irradiation accelerates apoptosis in human polymorphonuclear leukocytes: protection by LPS and GM-CSF. *Journal of Leukocyte Biology*.

[33] McClave J. T., Sincich T. T. (2012). *Statistics*.

[34] Homburg C. H., de Haas M., von dem Borne A. E., Verhoeven A. J., Reutelingsperger C. P., Roos D. (1995). Human neutrophils lose their surface Fc gamma RIII and acquire Annexin V binding sites during apoptosis in vitro. *Blood*.

[35] Luo H. R., Loison F. (2008). Constitutive neutrophil apoptosis: mechanisms and regulation. *American Journal of Hematology*.

[36] Akgul C., Moulding D. A., Edwards S. W. (2001). Molecular control of neutrophil apoptosis. *FEBS Letters*.

[37] Hakkim A., Fuchs T. A., Martinez N. E. (2011). Activation of the Raf-MEK-ERK pathway is required for neutrophil extracellular trap formation. *Nature Chemical Biology*.

[38] García-García E., Nieto-Castañeda G., Ruiz-Saldaña M., Mora N., Rosales C. (2009). Fc*γ*RIIA and Fc*γ*RIIIB mediate nuclear factor activation through separate signaling pathways in human neutrophils. *The Journal of Immunology*.

[39] Chuang F. Y. S., Sassaroli M., Unkeless J. C. (2000). Convergence of Fc*γ* receptor IIA and Fc*γ* receptor IIIB signaling pathways in human neutrophils. *Journal of Immunology*.

[40] Patel S., Kumar S., Jyoti A. (2010). Nitric oxide donors release extracellular traps from human neutrophils by augmenting free radical generation. *Nitric Oxide*.

[41] Lapponi M. J., Carestia A., Landoni V. I. (2013). Regulation of neutrophil extracellular trap formation by anti-inflammatory drugs. *Journal of Pharmacology and Experimental Therapeutics*.

[42] Kolaczkowska E., Kubes P. (2013). Neutrophil recruitment and function in health and inflammation. *Nature Reviews Immunology*.

[43] Futosi K., Fodor S., Mócsai A. (2013). Neutrophil cell surface receptors and their intracellular signal transduction pathways. *International Immunopharmacology*.

[44] Mayadas T. N., Cullere X., Lowell C. A. (2014). The multifaceted functions of neutrophils. *Annual Review of Pathology*.

[45] Urban C. F., Ermert D., Schmid M. (2009). Neutrophil extracellular traps contain calprotectin, a cytosolic protein complex involved in host defense against *Candida albicans*. *PLoS Pathogens*.

[46] Aleyd E., van Hout M. W. M., Ganzevles S. H. (2014). IgA enhances netosis and release of neutrophil extracellular traps by polymorphonuclear cells via FC*α* receptor I. *The Journal of Immunology*.

[47] Kelley J. M., Monach P. A., Ji C. (2011). IgA and IgG antineutrophil cytoplasmic antibody engagement of Fc receptor genetic variants influences granulomatosis with polyangiitis. *Proceedings of the National Academy of Sciences of the United States of America*.

[48] Pilsczek F. H., Salina D., Poon K. K. H. (2010). A novel mechanism of rapid nuclear neutrophil extracellular trap formation in response to *Staphylococcus aureus*. *The Journal of Immunology*.

[49] Neeli I., Radic M. (2013). Opposition between PKC isoforms regulates histone deimination and neutrophil extracellular chromatin release. *Frontiers in Immunology*.

[50] Van Ziffle J. A., Lowell C. A. (2009). Neutrophil-specific deletion of Syk kinase results in reduced host defense to bacterial infection. *Blood*.

[51] Newton A. C. (2008). Protein kinase C. *IUBMB life*.

[52] Popa-Nita O., Proulx S., Paré G., Rollet-Labelle E., Naccache P. H. (2009). Crystal-induced neutrophil activation. XI. Implication and novel roles of classical protein kinase C. *Journal of Immunology*.

[53] Jancinova V., Perecko T., Nosal R., Svitekova K., Drabikova K. (2013). The natural stilbenoid piceatannol decreases activity and accelerates apoptosis of human neutrophils: involvement of protein kinase C. *Oxidative Medicine and Cellular Longevity*.

[54] Keshari R. S., Verma A., Barthwal M. K., Dikshit M. (2013). Reactive oxygen species-induced activation of ERK and p38 MAPK mediates PMA-induced NETs release from human neutrophils. *Journal of Cellular Biochemistry*.

[55] Zhou M. J., Brown E. J. (1994). CR3 (Mac-1, *α*M*β*2, CD11b/CD18) and Fc*γ*RIII cooperate in generation of a neutrophil respiratory burst: requirement for Fc*γ*RIII and tyrosine phosphorylation. *The Journal of Cell Biology*.

[56] Byrd A. S., O'Brien X. M., Johnson C. M., Lavigne L. M., Reichner J. S. (2013). An extracellular matrix-based mechanism of rapid neutrophil extracellular trap formation in response to *Candida albicans*. *The Journal of Immunology*.

[57] Gillenius E., Urban C. F. (2015). The adhesive protein invasin of *Yersinia pseudotuberculosis* induces neutrophil extracellular traps via *β*1 integrins. *Microbes and Infection*.

[58] Branzk N., Lubojemska A., Hardison S. E. (2014). Neutrophils sense microbe size and selectively release neutrophil extracellular traps in response to large pathogens. *Nature Immunology*.

[59] Tosi M. F., Berger M. (1988). Functional differences between the 40 kDa and 50 to 70 kDa IgG Fc receptors on human neutrophils revealed by elastase treatment and antireceptor antibodies. *The Journal of Immunology*.

[60] Nagarajan S., Venkiteswaran K., Anderson M., Sayed U., Zhu C., Selvaraj P. (2000). Cell-specific, activation-dependent regulation of neutrophil CD32A ligand-binding function. *Blood*.

