# Supplementary material for: Differential Use of Human Neutrophil Fcγ Receptors for Inducing Neutrophil Extracellular Trap Formation
Source: J Immunol Res. 2016 Jan 14;2016:2908034. doi: 10.1155/2016/2908034 (PMC4806689; doi:10.1155/2016/2908034)
Supplement: Supplementary file 1 — The Fcgamma Receptor and Integrin expression on human neutrophils was confirmed with use of the corresponding monoclonal antibodies (Fig. 1S). Neutrophils and NETs are shown both in bright field and fluorescence images (Fig. 2S). NETs are decorated with histones (Fig. 3S) and with neutrophil elastase (Fig. 4S) as shown in these higher magnification (400 X) microphotographs. Neutrophils were not in apoptosis after FcgammaR crosslinking since they did not bind Annexin V (Fig. 5S). NETs induced by Fc gamma RIIIb crosslinking was not inhibited by the Syk inhibitor iSyk (Fig. 6S). Reactive Oxygen Species (ROS) formation was inhibited by the NADPH-oxidase inhibitor diphenyleneiodinium (DPI) (Fig. 7S). Fluorescent beads used in phagocytosis experiments were efficiently opsonized by anti FcgammaR-specific antibodies (Fig. 8S), and could be easily separated from neutrophils by flow cytometry (Fig. 9S). Phagocytosis was assessed the the appearance of cells with high fluorescence (Fig. 9S). Phagocytosis was also assessed by microscopy, calculating the Phagocytic Index, the number of beads ingested by 100 neutrophils (Fig. 10S). [file 2908034.f1.pdf]

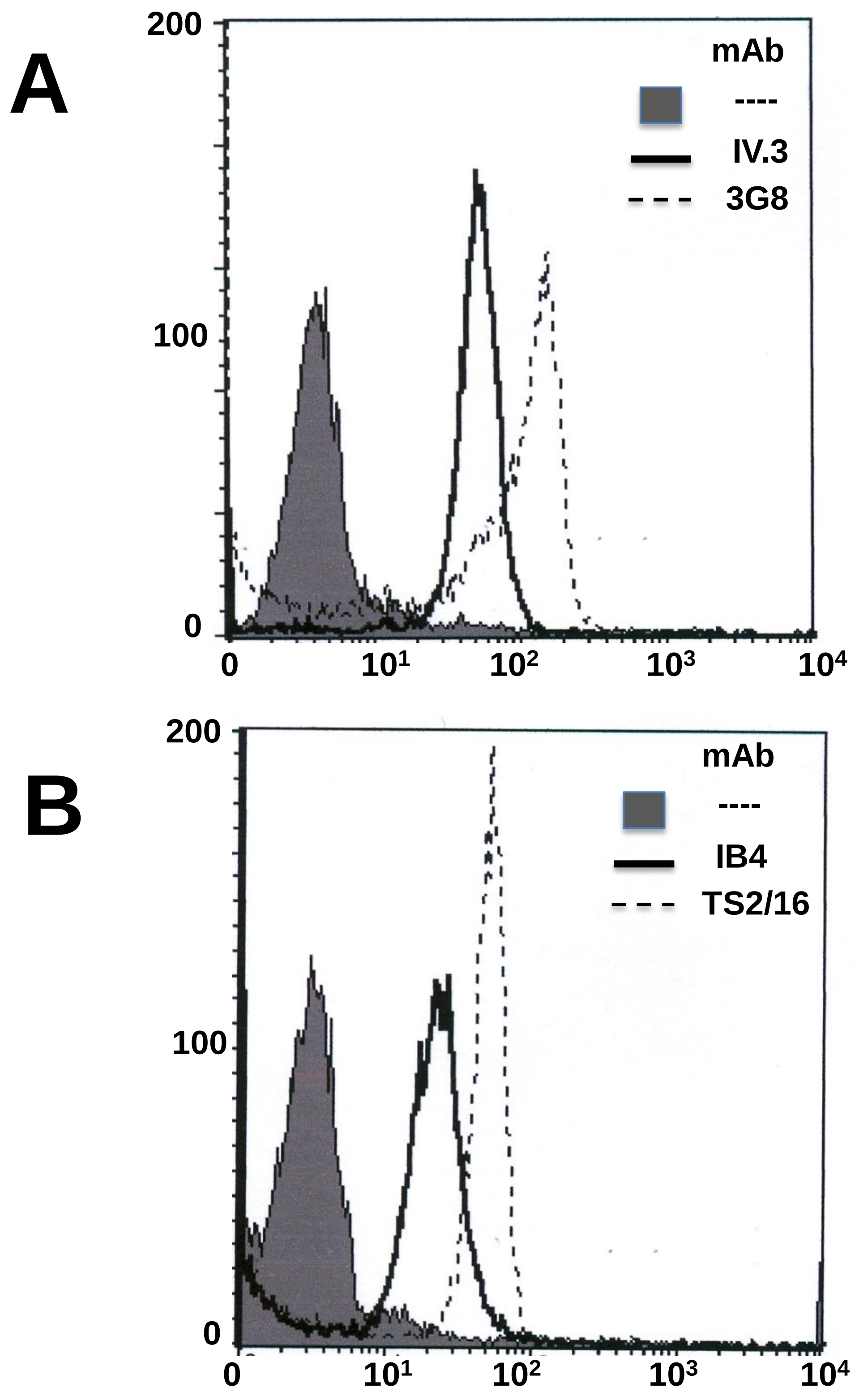
A
200
100
0
mAb

IV.3
3G8
0
10^1
10^2
10^3
10^4
B
200
100
0
mAb

IB4
TS2/16
0
10^1
10^2
10^3
10^4

Figure 1S

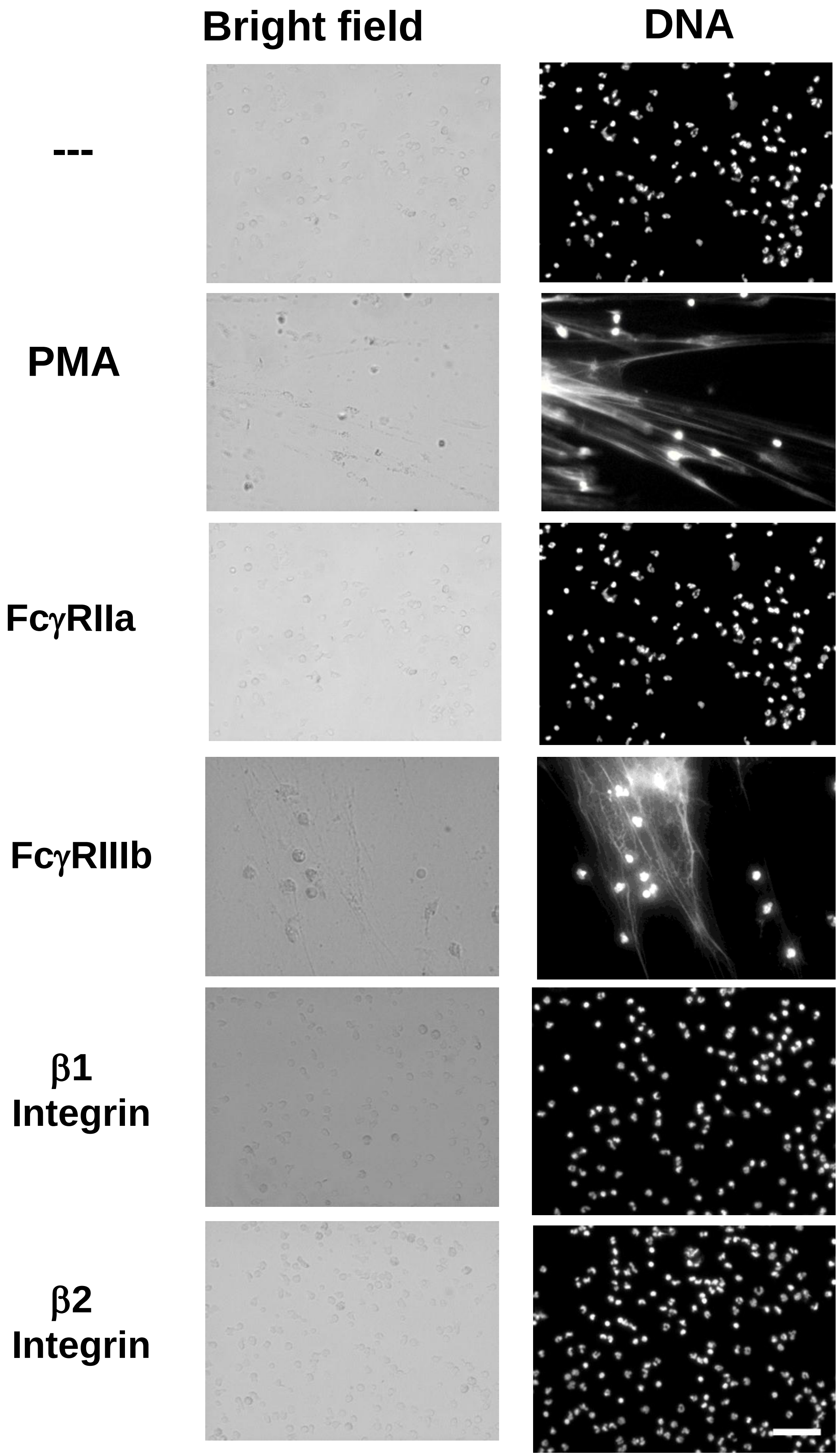
Bright field
DNA

PMA
FcγRIIa
FcγRIIIb
β1
Integrin
β2
Integrin

Figure 2S

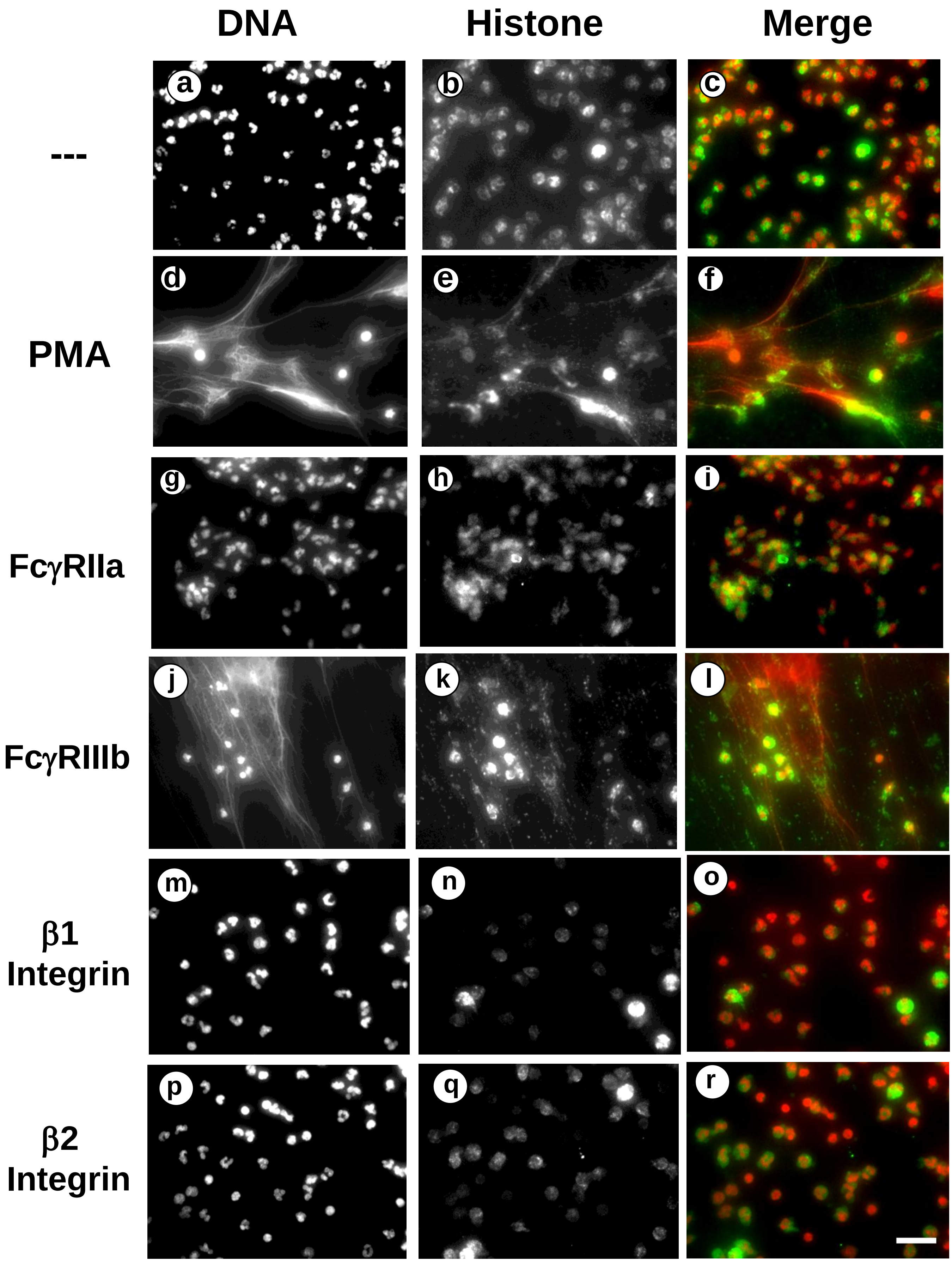
DNA
Histone
Merge

PMA
FcγRIIa
FcγRIIIb
β1
Integrin
β2
Integrin
a
b
c
d
e
f
g
h
i
j
k
l
m
n
o
p
q
r

Figure 3S

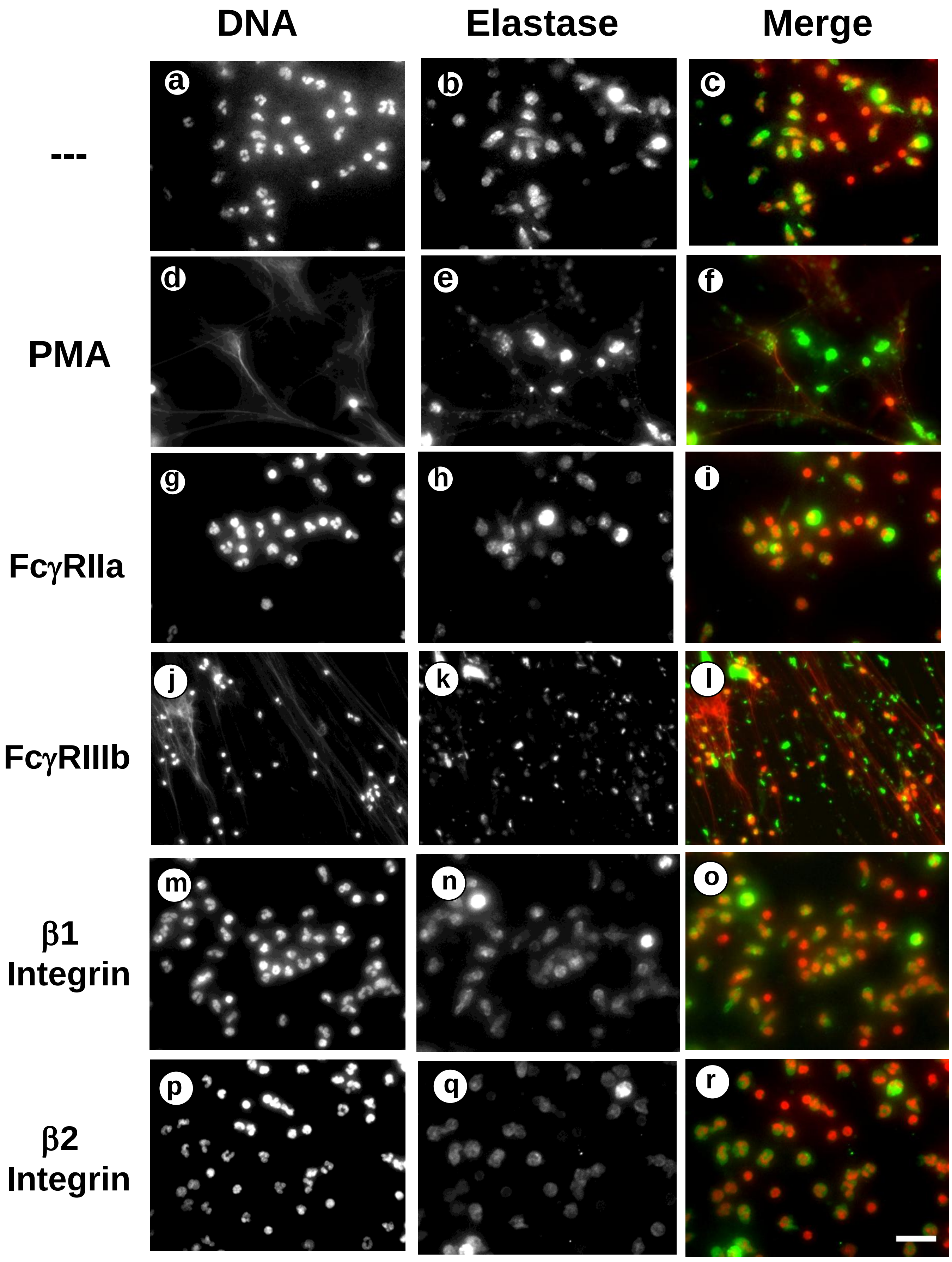
DNA
Elastase
Merge

PMA
FcγRIIa
FcγRIIIb
β1 Integrin
β2 Integrin
a
b
c
d
e
f
g
h
i
j
k
l
m
n
o
p
q
r

Figure 4S

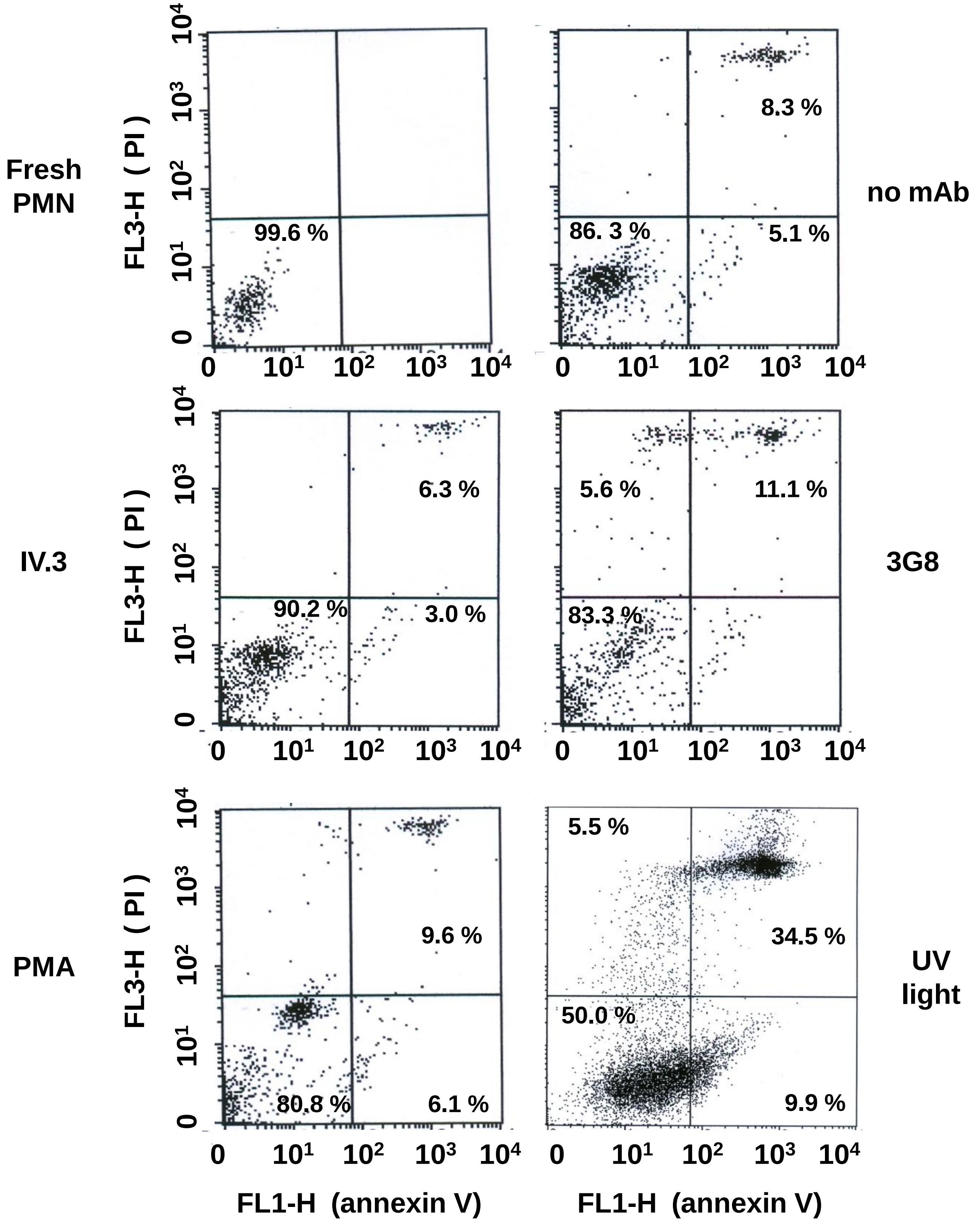
Fresh PMN
no mAb
IV.3
3G8
PMA
UV light
FL3-H (PI)
FL1-H (annexin V)
99.6 %
8.3 %
86. 3 %
5.1 %
6.3 %
90.2 %
3.0 %
5.6 %
11.1 %
83.3 %
9.6 %
80.8 %
6.1 %
5.5 %
34.5 %
50.0 %
9.9 %

Figure 5S

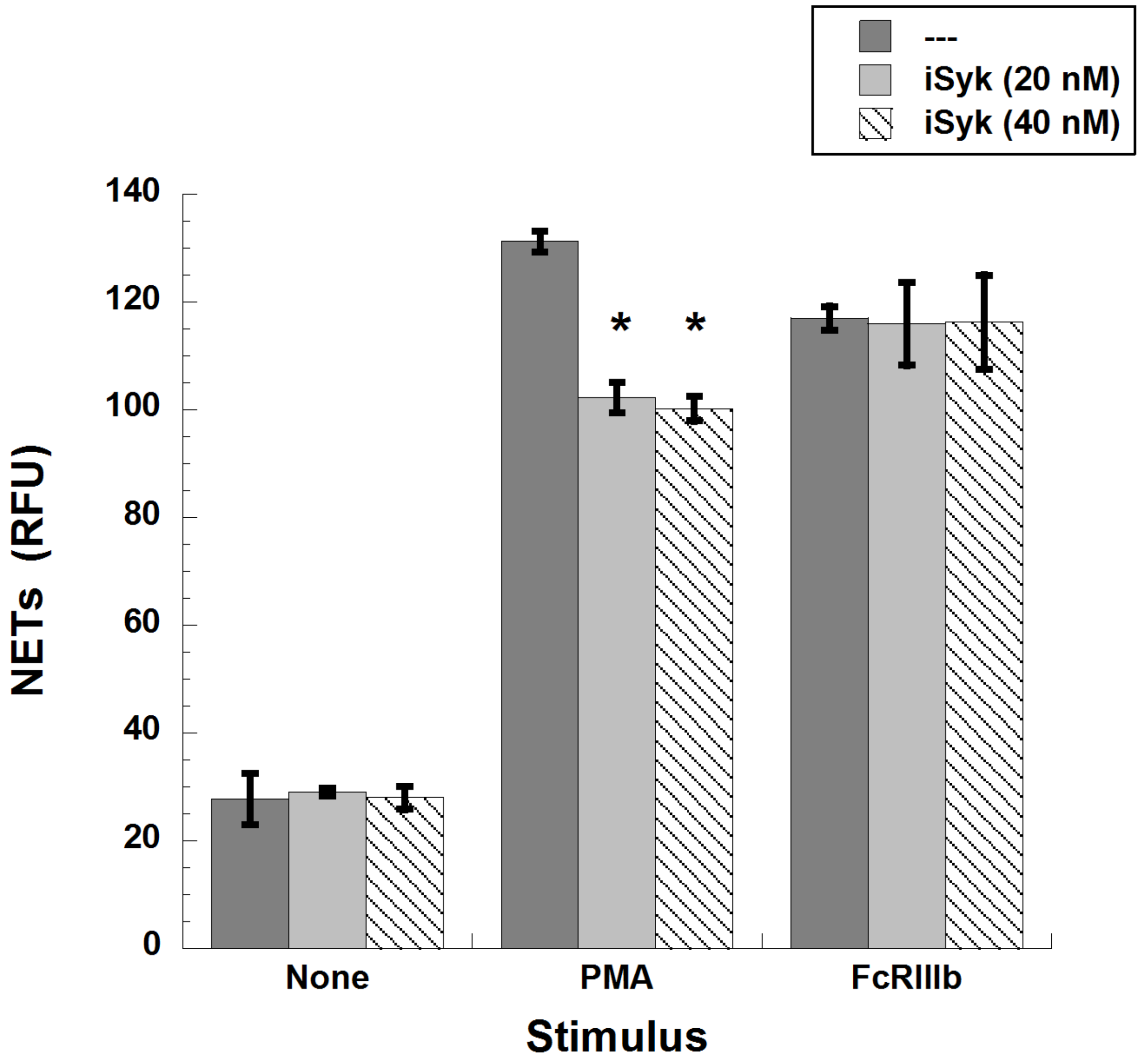
---
iSyk (20 nM)
iSyk (40 nM)
140
120
100
80
60
40
20
0
NETs (RFU)
*
*
None
PMA
FcRIIIb
Stimulus

Figure 6S

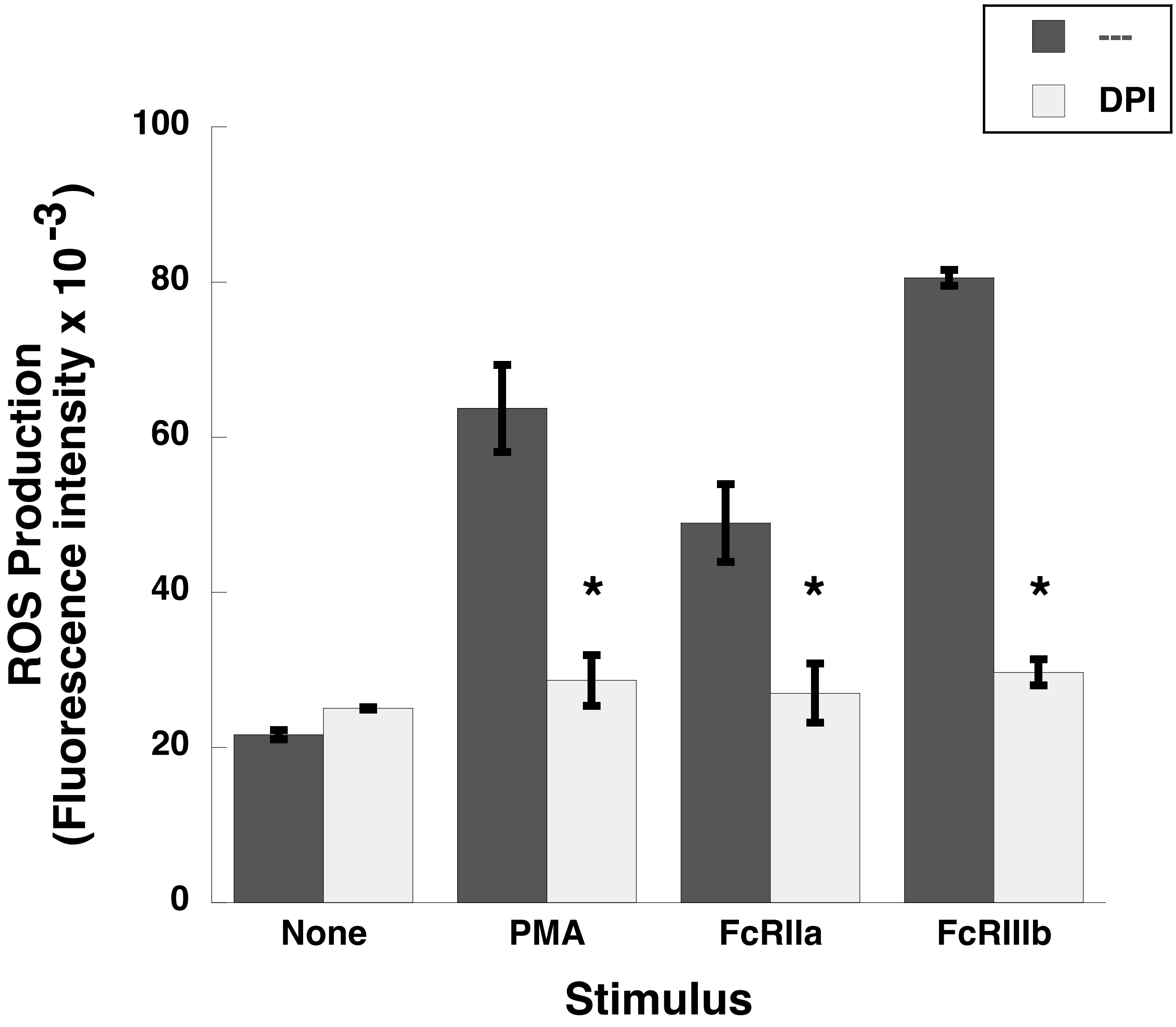

DPI
ROS Production
(Fluorescence intensity x 10^-3)
100
80
60
40
20
0
*
*
*
None
PMA
FcRIIa
FcRIIIb
Stimulus

Figure 7S

A

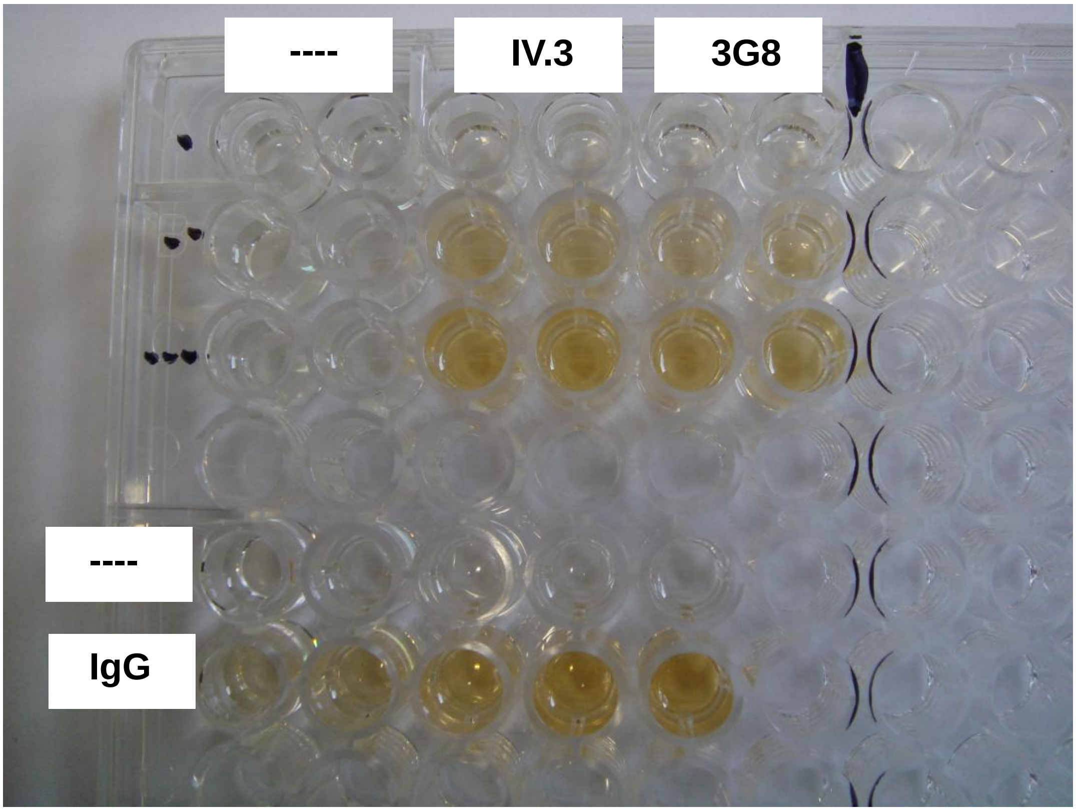

IV.3
3G8

IgG

B

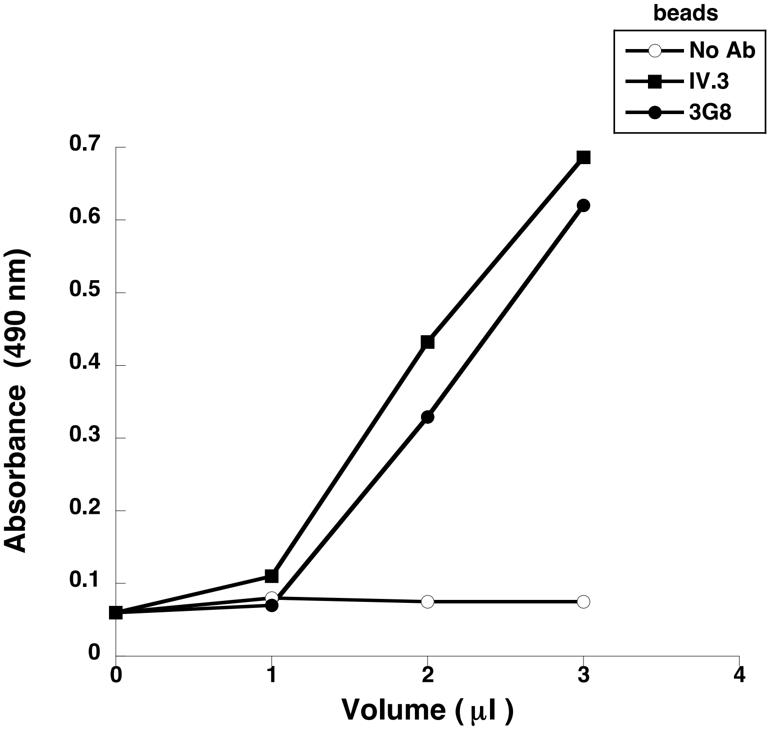
beads
No Ab
IV.3
3G8
Absorbance (490 nm)
0
0.1
0.2
0.3
0.4
0.5
0.6
0.7
0
1
2
3
4
Volume (μl)

Figure 8S

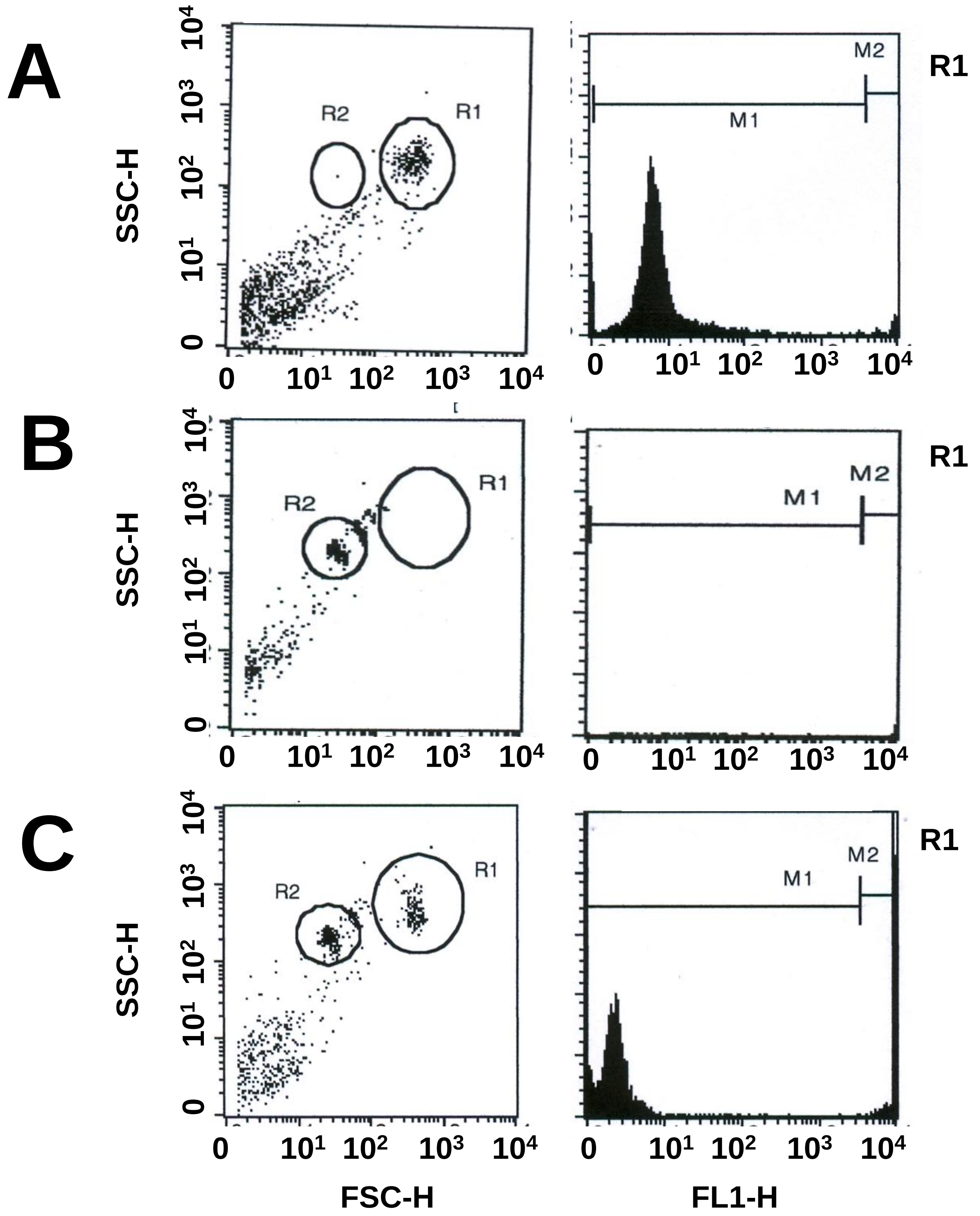

Figure 9S

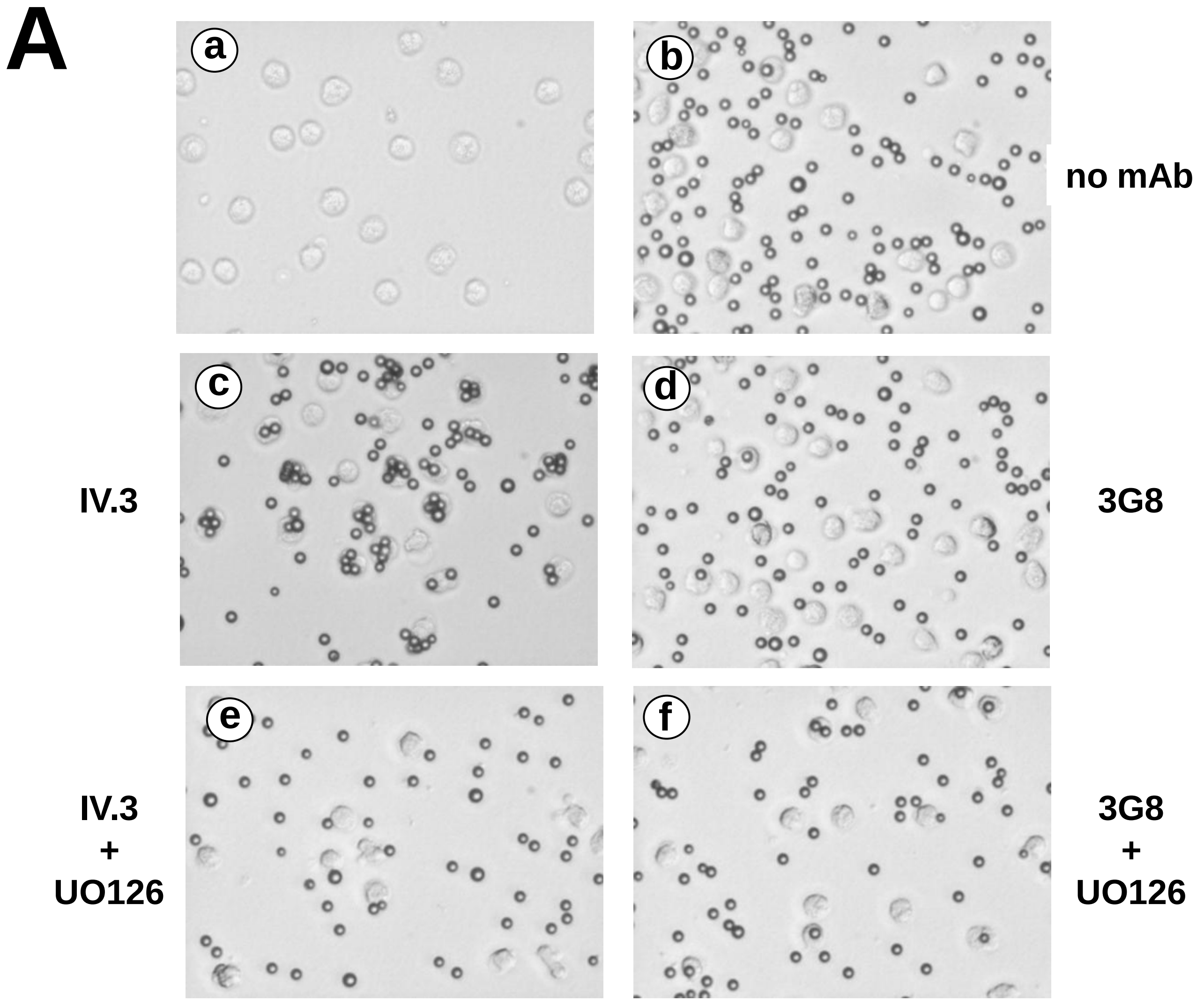
A
a
b
no mAb
c
d
IV.3
3G8
e
f
IV.3
+
UO126
3G8
+
UO126

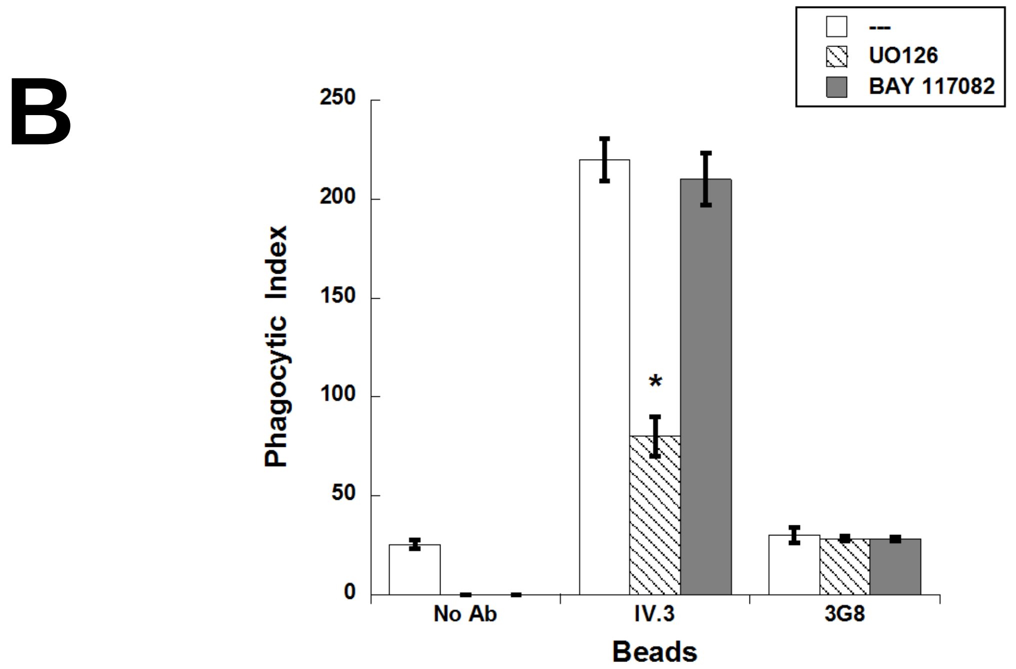
B

UO126
BAY 117082
Phagocytic Index
250
200
150
100
50
0
*
No Ab
IV.3
3G8
Beads

Figure 10S
